# Therapeutic Bioactivity Exerted by the *Apis mellifera* Bee Venom and Its Major Protein Melittin: A Scoping Review

**DOI:** 10.3390/molecules30194003

**Published:** 2025-10-07

**Authors:** Perihan Mutlu Erdoğan, Funda Bilgili-Tetikoğlu, Selcen Çelik-Uzuner, Oktay Yıldız, Sevgi Kolayli, Dimitris Mossialos

**Affiliations:** 1 Department of Molecular Biology and Genetics, Faculty of Science, Karadeniz Technical University, 61080 Trabzon, Türkiye; dinaraerdogan@gmail.com (P.M.E.); fundabilgili@ktu.edu.tr (F.B.-T.); selcen.celik@ktu.edu.tr (S.Ç.-U.); 2 Graduate School of Natural and Applied Science, Karadeniz Technical University, 61080 Trabzon, Türkiye; 3 Faculty of Pharmacy, Karadeniz Technical University, 61080 Trabzon, Türkiye; oktayyildiz@ktu.edu.tr; 4 Trabzon Teknocity, Okta R&D Eng Services Industry Trade Limited Company, 61080 Trabzon, Türkiye; 5 Gumushane University, Rectorate, 29100 Gümüşhane, Türkiye; 6 Department of Chemistry, Faculty of Science, Karadeniz Technical University, 61080 Trabzon, Türkiye; skolayli61@yahoo.com; 7 Microbial Biotechnology-Molecular Bacteriology-Virology Laboratory, Department of Biochemistry & Biotechnology, University of Thessaly, 41500 Larissa, Greece

**Keywords:** bee venom, melittin, anti-cancer, anti-microbial, anti-inflammation, immunomodulatory effect, anti-oxidant

## Abstract

Honey bee (*Apis mellifera*) products have been extensively utilized in traditional medicine. Bee venom (BV) is one of the major bee products with a high concentration of the small peptide melittin (MEL) and exerts bioactivity ranging from anti-microbial to anti-inflammatory and anti-cancer. This scoping review aims to sum up research articles on the bioactivity exerted by BV and MEL published in PubMed and Scopus from 2010 onwards. PRISMA guidelines were implemented to analyze the relevant literature; we ended up with 425 research articles. Bioactivity of BV and MEL was grouped as (i) anti-inflammatory (85), (ii) immunomodulatory (37), (iii) anti-microbial (179), (iv) anti-cancer (170), and (v) anti-oxidant (32). Although there is a significant body of research on the anti-cancer and anti-microbial activity of BV and MEL, their anti-oxidant, anti-inflammatory and immunomodulatory properties have received comparatively less attention. Many studies on the immunomodulatory effects of BV or MEL have focused on cancer. However, the effects on Parkinson’s and Alzheimer’s disease have not been extensively studied regarding the anti-inflammatory effects. Given the critical role of the immune system and inflammatory response in cancer, neurodegenerative diseases, senescence and against infections, it is paramount to further explore the immunomodulatory and anti-inflammatory potential of BV and MEL.

## 1. Introduction

Bee venom (BV), also known as apitoxin, is a natural mixture produced in the venom glands of bees belonging to the order Hymenoptera, which are the type of insects that generally sting their adversaries to protect themselves [1,2]. Honey bees belong to the genus *Apis* and are important crop pollinators, especially *Apis mellifera*, the European Honey Bee [1]. BV has a long history as a therapeutic substance for thousands of years, from ancient Egypt to China [2,3]. Honey bee venom is a mixture of bioactive substances such as peptides, proteins, enzymes, and other small molecules in an odorless, translucent, acidic liquid form with an average pH of 5 [4,5,6]. It is estimated that BV contains more than 200 different compounds, and more than half of them are quantified [7]. BV is harvested from honeybees with continuous electro-simulation according to the Benton protocol [8]. The most abundant BV compound is the peptide melittin (MEL), which makes up approximately 60% of dry BV weight, varying between different bee species [9,10]. The enzyme phospholipase A2 (PLA2) makes up to 20% of dry BV weight (Table 1). Another peptide, apamin, is the third most abundant compound, though it makes up to only 1% of dry BV weight, followed by approximately 200 less abundant molecules (Table 1) [7,9,10]. Numerous in vivo and in vitro studies have demonstrated that BV exerts strong bioactivity, such as anti-cancer, anti-nociceptive, anti-oxidant, anti-bacterial, anti-fungal, anti-viral, anti-inflammatory, anti-arthritic, anti-metastatic, and hemolytic effects [5,6,11,12]. Especially in East Asia, BV is used for acupuncture to treat chronic pain, generally caused by inflammation or neuropathy [13,14].

## 2. Methodology

This scoping review focuses on the potentially therapeutic bioactivity exerted by the bee venom and its compounds, especially melittin, as described in original research articles. Our main goal was to implement the PRISMA guidelines to summarize and categorize the relevant scientific literature, to identify research gaps, and to suggest future perspectives on research relevant to the bioactivity exerted by BV and MEL.

The widely used Scopus and PubMed databases were searched in this review. The last search took place on 28 May 2025. Since both sites use different ID systems (EID for Scopus, PMID for PubMed), DOI links were used to store and compare the data of the documents obtained through browsing. According to our expertise on the topic, two search groups were formed, namely BV and MEL. We included general keywords to combine BV and MEL with specific types of bioactivities (Table 2). The keywords that were suggested by the databases were also considered (Table 2).

In PubMed, the search filter “best match” was applied, while in Scopus, it was “relevance”. Published articles from 2010 onwards were included. In Scopus, language was filtered with English; however, since PubMed is a US-based website, no language filter was applied. Original research and review articles were prioritized, and some literature items were considered not eligible, especially letters formatted as “letter to the editor, correction, correspondence,” etc., which don’t focus on detailed, complete research. Zotero was used as an automation program to recognize the documents and to remove duplicate results. The articles were read, categorized, and presented in tables and figures, according to their type and content.

**Figure 1:**
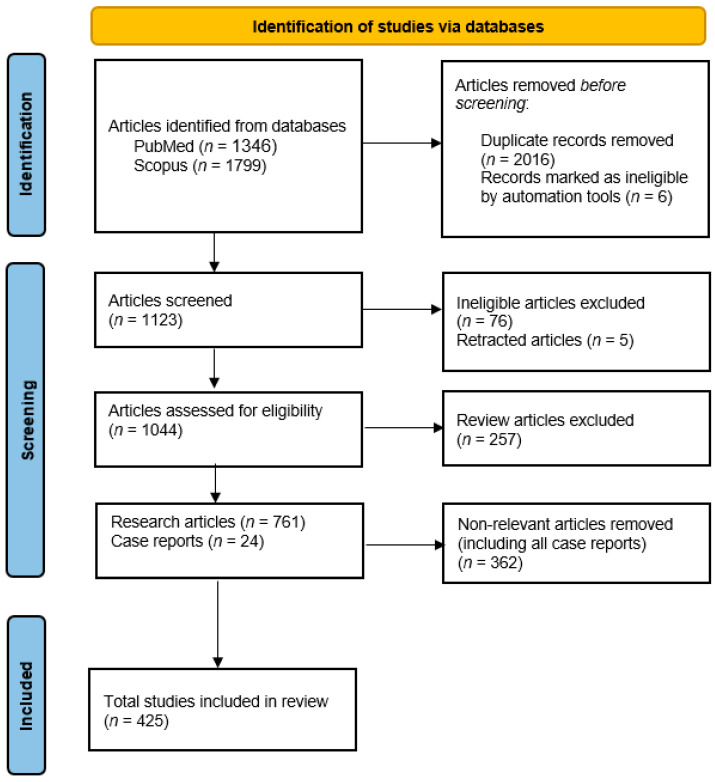
PRISMA flowchart: Implementation of PRISMA guidelines followed during this review in order to include eligible studies [16].

The overall search yielded a total of 3145 items. After the 2016 duplicates and 6 publications that were marked unidentified by the automation tool were removed using Excel and Zotero, 1123 articles remained. After reviewing, 76 articles were removed because they were not original research or review articles. These publications were either letters to the editor, communications, corrections or editorials. Also, 5 articles were retracted, leaving 1044 articles for assessment for eligibility. After reviewing, the remaining 788 articles were identified as 761 research articles and 24 case reports. 425 articles were found to be relevant to our topic (Figure 1).

The remaining articles were categorized based on their type of bioactivity. BV and MEL are both used in different types of in vivo studies (clinical and animal models) as well as in vitro (cell lines, microorganisms). Types of animal models (generally rats or mice), along with administration types (injection, topical, acupuncture), targeted microorganisms, cell lines and delivery systems are listed in the experimental design section. The “additional information” column in the table provides other types of bioactivity or other specific information regarding the experimental design. It also lists the types of substances used—such as bee venom (BV), melittin, and other components of BV—along with their sources, including *Apis mellifera* (honeybee), general references to bees, or other species. Overall, regarding the bioactivity described or studied, the relevant articles were categorized as follows: (i) anti-inflammatory, (ii) immunomodulatory, (iii) anti-microbial, (iv) anti-cancer, and (v) anti-oxidant and they are presented below.

## 3. Content of Bee Venom

**Figure 2:**
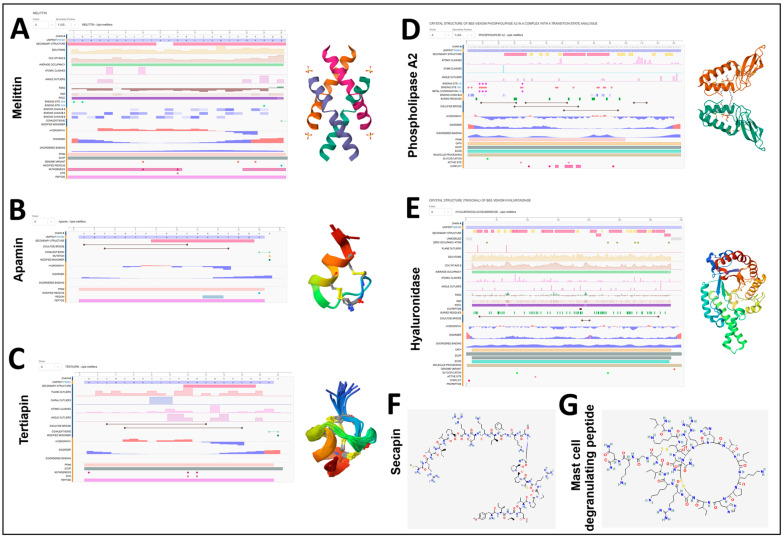
Chemical structure of major bee venom compounds: (**A**–**G**) show the secondary and 3D structures of melittin, apamin, tertiapin, phospholipase A2, hyaluronidase, secapin, and mast cell degranulation peptide, respectively. (**A**–**E**) Domain organization, sequence conservation, and predicted physicochemical features of principal bee venom proteins visualized using bioinformatics tools. (**A**) Melittin, a 26-amino-acid amphipathic peptide responsible for hemolytic and cytolytic activities, shows a characteristic α-helical conformation. (**B**) Apamin, a small neurotoxic peptide that blocks Ca^2+^-activated K^+^ channels, displays a compact disulfide-stabilized α-helical structure. (**C**) Tertiapin, known for its potassium channel inhibitory activity, presents a short α-helix with disulfide bridges. (**D**) Phospholipase A_2_ (PLA_2_), the primary enzymatic component of bee venom, possesses conserved catalytic and Ca^2+^-binding domains, which contribute to membrane degradation and inflammation. (**E**) Hyaluronidase, often termed the “spreading factor,” shows conserved glycosidic hydrolase motifs and facilitates venom diffusion through connective tissues. (**F**,**G**) Three-dimensional molecular structures of smaller venom-derived peptides, including Secapin (**F**) and the Mast Cell Degranulating (MCD) peptide (**G**), highlight the presence of loop and β-turn motifs important for their antimicrobial and immunomodulatory activities. Structural models were generated using protein databases and homology modeling algorithms to visualize functional domains and peptide folding patterns (obtained from pubchem.ncbi.nlm.nih.gov and www.rcsb.org databases).

Bee venom consists of a range of bioactive molecules. Bee venom compounds, including melittin, apamin, secapin, mast cell degranulating (MCD) peptide, tertiapin, hyaluronidase, and phospholipase A2, act synergistically, contributing to the overall bioactivity exerted by bee venom, inducing cytolytic, neurotoxic, pro-inflammatory, allergenic, and anti-microbial effects. The most bioactive compound, Melittin (MEL), is a small peptide of 26 amino acid residues with a weight of 2840 Da (GIGAVLKVLTTGLPALISWIKRKRQQ-CONH2) and is often described as an “anti-microbial peptide (AMP)” (Figure 2A). Alongside the asymmetrical distribution of polar and non-polar amino acid residues, melittin structure demonstrates an amphipathic nature, therefore making it a highly bioactive component, especially against membranes. Therefore, melittin disrupts the membranes of cancer cells, bacteria, and fungi. In many in vitro experiments, melittin is used as a positive control for such effects. Furthermore, melittin blocks metabolic pathway elements, including Tumor Necrosis Factor (TNF) receptors, cytokine signaling pathways, and Epidermal Growth Factor Receptor (EGFR), thus suggesting its role in promoting apoptosis [1,2,3,4]. **Apamin** is a neurotoxin that crosses the blood–brain barrier. It contains 18 amino acid residues with two disulfide bonds (Cys1-Cys11, Cys3-Cys15) in between (Figure 2B). Since animal venom toxins are often modified post-translationally, apamin C-terminal is amidated. In the early 1980s, apamin was used to identify certain Ca^2−^ activated K ion channels due to its selective activity towards these channels. Moreover, it is known to demonstrate antimicrobial effects. Due to molecular stability, apamin is a possible drug delivery element. **Tertiapin** (Figure 2C) is a 21-amino-acid peptide that blocks potassium channels [5,6,7,8]. **Phospholipase A2** from bee venom (BvPLA2) is a type of secreted PLA2. BvPLA2 is Ca^2+^-dependent, catalyzing the hydrolysis of sn-2 acyl phospholipid bonds often implicated in inflammation, leading to the production of lysophosphatidic acid and arachidonic acid (Figure 2D). However, these interactions also exert anti-inflammatory activity from a medical perspective. Similarly to melittin, it plays a role in membrane disruption [5,9,10]. **Hyaluronidase** (Figure 2E) promotes allergenic response. It cleaves hyaluronan of the connective tissue and accelerates absorption of the bee venom into the tissue [5,11]. **Secapin** (Figure 2F) is a potential neurotoxin with anti-fibrinolytic, anti-elastolytic, and anti-microbial effects [5,6,7,8]. **Mast Cell Degranulating (MCD) peptide** has a similar structure to apamin (Figure 2G) and might also act like a neurotoxin. At low concentrations, it induces mast cell degranulation; at high concentrations, it has anti-inflammatory effects.

## 4. Bioactivity Exerted by Bee Venom and Melittin

### 4.1. Anti-Inflammatory Effects

#### 4.1.1. Anti-Inflammatory Bioactivity Targeting Various Diseases

Anti-inflammatory effects of BV and MEL targeting diverse diseases are a popular research topic. Both BV and MEL are used as therapeutic tools against diseases where inflammation is a major issue mediating morbidity, most of them being neurological or immunological disorders (Table 3). Much research is focused on BV and MEL effects on inflammatory cytokine pathways and gene expression related to inflammation and immunomodulation. Both chronic and acute diseases are subjects of anti-inflammatory action exerted by BV and MEL. Animal in vivo studies are the most common experimental design.

Despite extensive evidence supporting the anti-inflammatory and therapeutic potential of bee venom (BV) and its components like melittin, apamin, and PLA2 across diverse disease models, several research gaps remain. Most studies are limited to preclinical models, with scarce clinical trials validating safety, efficacy, dosing, and delivery mechanisms in humans. Furthermore, the mechanisms underlying BV’s immunomodulatory and anti-oxidant effects require deeper molecular investigation, especially in chronic and complex diseases. Standardization of BV extraction, formulation, and administration methods is lacking, which hinders reproducibility and clinical translation. Future research should focus on large-scale, controlled clinical trials, advanced drug delivery systems (e.g., nanoparticles, hydrogels), and long-term toxicity studies. Additionally, exploring BV’s synergistic effects with conventional therapies and its potential against antibiotic resistance and neurological disorders warrants further exploration.

#### 4.1.2. Rheumatoid Arthritis

Rheumatoid arthritis (RA) is a conspicuous disease target of BV (as well as its compounds) therapy (Table 4). It is a chronic inflammatory autoimmune disease that manifests as pain all over the body and may escalate into permanent loss of function, mobility issues, and a dramatic decrease in the quality of life [75]. MEL and PLA2 anti-inflammatory therapeutic effects are hot research topics of traditional medicine (especially traditional Korean medicine), and acupuncture/acupoint injection is commonly practiced with animal and human clinical models.

Most findings are limited to animal models or in silico analyses, with minimal translation into well-designed clinical trials. Delivery methods such as nanoparticles, microneedles, and liposomes are emerging but require optimization for consistent dosing, safety, and patient compliance. Future research should prioritize mechanistic studies to elucidate molecular targets, long-term safety assessments, and randomized controlled trials in humans. Moreover, integrating nanotechnology-based delivery systems with immunomodulatory strategies may enhance therapeutic outcomes in RA management.

#### 4.1.3. Non-Disease Targeted Anti-Inflammatory Activity

Rather than targeting diseases, many researchers are looking into the potentially protective aspects of BV/melittin, which involve pathways and membrane interactions. Some are in vivo with rats and mice, but some are in vitro studies using cell lines and microorganisms (Table 5).

The immune system evolved in multicellular organisms in order to fight infections and other disorders such as cancer. By modulating immunological responses, such as T cell and macrophage activation, certain immunological pathways help to combat diseases. Both BV and melittin are known for their immunomodulatory effects due to their bioactive potential (Table 6). Nevertheless, another less abundant BV compound, PLA2, is described to exert immunomodulatory activity in BV-related studies.

Bee venom and its components, such as melittin and PLA2, show strong immunomodulatory and anti-inflammatory effects across a range of non-cancerous conditions including autoimmune, respiratory, and neurological diseases. However, several research gaps are evident. Many studies are preclinical, lacking human trials that could confirm efficacy, safety, and optimal dosing. The long-term effects of repeated BV exposure, particularly regarding immune tolerance or hypersensitivity, are not well understood. Furthermore, while novel delivery systems (e.g., nanoparticles, microneedles, LNPs) are emerging standardized methods for formulation and administration are still underdeveloped. Future research should focus on (i) well-controlled clinical studies, (ii) elucidation of mechanistic insights regarding immune regulation pathways, and (iii) the development of safe, targeted delivery platforms to minimize toxicity and maximize therapeutic outcomes.

Cancer therapy is often associated with adverse effects and might be inefficient in certain cancer types. Minimizing the side effects of chemotherapy is an active research topic that explores many alternative therapy options, such as apitherapy. Immunomodulatory effects might be considered either enhancing (immunostimulation) or suppressing (immunosuppression) [108]. Immunomodulatory agents can alter the functions of immune cells, regulate cytokine production, or affect inflammatory responses. As a result, immunomodulation has emerged as a significant therapeutic target, particularly in the treatment of cancer, autoimmune diseases, and chronic inflammation. Natural compounds and their derivatives are of interest in immunomodulation interventions [109]. Bee venom and melittin have attracted increasing attention in recent years due to their immunomodulatory properties (Table 7). These natural products might promote immune response in immunocompromised patients or control excessive immune responses by restoring immune balance, thus leading to more efficient cancer therapy.

### 4.2. Anti-Microbial Activity

Anti-microbial resistance (AMR) is an escalating global crisis. AMR is driven by the overuse and misuse of antibiotics, leading to the rise in healthcare-associated infections worldwide. It is projected that by 2050, 10 million people annually may die from infections that cannot be treated due to resistant bacteria and the ineffectiveness of current antibiotics [127]. In that respect, alternatives to classic antibiotics and anti-fungal agents are constantly investigated, and the use of anti-microbial peptides (AMPs) is one of them. These small molecules are abundant as part of the innate immunity in many insects, including *Apis mellifera* and other Apis and non-Apis types of bees [128,129]. Considering that AMPs are small amino acid chains that don’t require complex protein folding mechanisms, AMP synthesis in heterologous hosts such as bacteria or fungi is feasible, thus leading to therapeutic applications [130]. Melittin, the major protein detected in BV, is a well-studied bacterial membrane disruption agent, and it is often used as a reference in studies related to other AMPs. The anti-microbial activity of MEL and BV against diverse multidrug-resistant pathogens such as *Staphylococcus aureus* (*S. aureus*), *Klebsiella pneumoniae* (*K. pneumoniae*), *Escherichia coli* (*E. coli*), *Pseudomonas aeruginosa* (*P. aeruginosa*), and *Candida albicans* (*C. albicans*) is well studied. Numerous studies describing these results are presented in Table 8, whereas often melittin is referred to as an Anti-Microbial Peptide (AMP). Overall, the in vitro antimicrobial activity of BV and especially MEL has been sufficiently studied. However, there is scarce data regarding the anti-biofilm effect of BV and MEL, the attenuation of virulence factors of diverse pathogens, and especially the response of pathogens to BV and MEL using OMICS technologies (for example, RNA-sequencing). Future research should be focused on more preclinical studies regarding in vivo validation and therapeutic potential, as well as clinical trials to assess efficacy, feasibility, and optimal dosage.

### 4.3. Anti-Cancer Activity

Alternatives for cancer therapy search for the minimum side effects for the patients. The goal is to maintain the health of the non-cancerous tissue while eliminating cancerous cells; therefore, selectivity. Traditional methods of chemotherapy suffer from inducing toxic effects, aforementioned acute kidney injury, which further deteriorates the odds of patient survival. In addition, MDR (multi-drug resistance) is another reason for alternative approaches [139,213].

Cytotoxic effects and BV and melittin are directly responsible for the therapeutic potential for anti-cancer research [295,296,297,298,299,300]. As mentioned before, the anti-microbial effect of BV and melittin relies on membrane interactions and the subsequent cytotoxicity. Mammalian cancers are much more complex threats to overcome due to their resistance to follow the cell cycle, eventually cell death. By size and membrane composition, mammalian cells are different from microorganisms; therefore, a small molecule in the complexity of BV may interfere with several pathways generally overexpressed in various cancer types. With this possibility, many studies are focused on gene expression and pathways in cancers. But this exact cytotoxic potential clashes with the goal of selectivity. To reduce the cytotoxic and hemolytic activity against healthy tissue; deliveries of both BV and melittin are considered and designed extensively in obtained research articles.

The cytotoxic effect of bee venom and melittin has been shown in both human and murine cancer and a variety of cancer cells such as breast [300], hepatocellular [301], cervical [302], pancreatic [303], colorectal cancers [304], glioblastoma [88,305] and melanoma [306], and also in rat or mice animal models of cancer [125,307,308] (Table 9 and Table 10). The anti-cancer effects of bee venom and melittin are given separately.

#### 4.3.1. Bee Venom in Cancer Research

The anti-cancer activity of bee venom (BV) has been studied in many cell lines and cancer types, alongside xenograft animal models (Table 9). The interactions of bee venom with pro-apoptotic pathways are a common study goal among these studies [256,309,310,311]. Delivery systems implementing nanoparticles are popular since BV may cause adverse effects such as unwanted hemolytic activity or allergic reactions [312,313].

**Table 9:** Cancer research with bee venom.

Article Name	Substance	Cancer Type	Experimental Design	Additional Information
(G. B. Jung et al., 2018) [295]	BV	Breast cancer	In vitro; MDA-MB-231, PBMLs cell lines	Anti-cancer effect
(A. A. Nagy et al., 2022) [296]	BV	Hepatocellular carcinoma	In vivo; Animal: rats Nano delivery of BV with iron oxide	Gene Expression-Pathway
(Sevin et al., 2023) [88]	BV (*Apis m.*) Apamin Melittin PLA2	Glioblastoma	In vitro; U87MG cell line	Cytotoxicity Gene expression-pathway Immunomodulation
(Yaacoub et al., 2022) [297]	PLA2, Melittin from BV (*Apis m.*)	Cervical cancer	In vitro; HeLa cell line	Cytotoxicity, anti-coagulation, proteolytic activity
(M. Sharaf et al., 2024) [209]	Nanoparticles loaded with apitoxin from BV (*Apis m.*)	Hepatocellular carcinoma Colon cancer	In vitro; HepG2, Caco-2, Vero cell lines Nano delivery of BV by chitosan coating	Cytotoxicity Anti-microbial
(H. N. Lim et al., 2019) [306]	Melittin from BV	Melanoma cancer	In vitro; B16F10, A375SM, SK-MEL-28 cell line	Gene Expression-Pathway, Cell invasion inhibition
(Mansour et al., 2021) [314]	BV (*Apis m.*) and melittin	Hepatocellular carcinoma	In vitro; HepG2, THLE-2 cell lines In silico; Molecular docking	Cytotoxicity, Gene Expression-Pathway
(Małek et al., 2022) [315]	BV (*Apis m.*)	Glioblastoma	In vitro; 8-MG-BA, GAMG, HT22 cell lines	Neurological, cytotoxicity, MMP-2 and MMP-9
(Tetikoğlu & Çelik-Uzuner, 2023) [316]	BV (*Apis m.*)	Breast cancer, Hepatocellular carcinoma	In vitro; MDA-MB-231, HepG2, NIH3T3 cell line	Genotoxicity, Gene Expression-Pathway
(Saghi et al., 2022) [309]	BV (*Apis m.*)	Colorectal cancer	In vitro; HT-29, NIH3T3 cell lines	Apoptosis Gene Expression-Pathway, ROS, anti-tumor
(D.-H. Kim et al., 2020) [256]	BV (*Apis m.*)	Cervical cancer	In vitro; C33A, HeLa, Caski cell line	Apoptosis, Gene Expression-Pathway
(J. Zhao et al., 2022) [310]	BV	Pancreatic cancer	In vitro; PANC-1 cell line	Apoptosis, Cytotoxicity, anti-metastatic
(J. E. Yu et al., 2022) [311]	BV	Lung cancer Glioblastoma, Hepatocellular carcinoma Breast cancer	In vitro; A549, A172, NCI-H460, MDA-MB-231, Hep3B cell lines	Apoptosis, Gene Expression-Pathway, autophagy
(Pinto et al., 2024) [32]	BV (*Apis m.*)	Hepatocellular carcinoma, Colon cancer, Breast cancer, Cervical cancer, Gastric adenocarcinoma	In vitro; HeLa, Caco-2, Vero, MCF-7, NCI-H460, AGS, PLP2, RAW 264.7, cell lines Nanoparticle delivery of BV	Anti-inflammatory, Therapeutic, Cytotoxicity
(Małek et al., 2025) [317]	BV (*Apis m.*), Melittin	Glioblastoma	In vitro: MO3.1, LN229 and LN18 cell line	Cytotoxicity
(Jeong et al., 2019) [318]	BV (*Apis m.*)	Lung cancer	In vitro: A549, H793, H23 lung cancer cell lines	Anti-metastatic, cytotoxicity, cell invasion inhibition, Gene expression pathway
(İlhan et al., 2025) [319]	BV	Thyroid medullary carcinoma	In vitro: Thyroid cancer cell line Nanoparticle delivery of BV: ZIF-8	Cytotoxicity, gene expression-pathway
(Amer et al., 2025) [320]	BV (*Apis m.*)	Lung cancer	In vitro: Vero, A549 cell line; bacteria: *Mycobacterium smegmatis* Nanoparticle delivery by chitosan	Anti-bacterial Anti-tumor Antibiotic resistance
(Halici et al., 2025) [321]	BV	Chronic myeloid leukemia (CML)	In vitro: K562 cell line	Cell viability
(Qanash et al., 2025) [322]	BV (*Apis m.*)	Hepatocellular Carcinoma	In vitro: HEPG2 cell line; Bacteria: *S. aureus*, *Bacillus subtilis*, *E. coli*, *Salmonella* Typhimurium; Fungi: *Aspergillus niger*, *C. albicans* Nanoparticle delivery by zinc oxide and polyvinyl alcohol nanofilm	Anti-inflammatory, Anti-microbial, cytotoxicity Anti-oxidant activity hemolytic activity
(Gülmez et al., 2017) [213]	BV (*Apis m.*)	Colon adenocarcinoma Cervical cancer, Glioblastoma	In vitro; HT-29, HeLa, C6, Vero	Anti-microbial, apoptosis, apitherapy, cytotoxicity
(Abdel-Monsef et al., 2023) [215]	Superoxide dismutase from BV (*Apis m.*)	Breast cancer Hepatocellular carcinoma	In vitro; HepG-2, MCF-7 cell lines	Anti-microbial, Characterization of BV, anti-tumor
(Sobral et al., 2016) [323]	BV (*Apis m.*)	Lung cancer Breast cancer Cervical cancer Leukemia Hepatocellular carcinoma	In vitro; HeLa, NCI-H460, RAW264.7, HepG2, MCF-7 cell lines	Anti-inflammatory, cytotoxicity, Anti-oxidant activity, Characterization of BV
(El Mehdi et al., 2021) [324]	BV (*Apis m.* intermissa)	Lung cancer Hepatocellular carcinoma Cervical cancer Melanoma Breast cancer	In vitro; HeLa, NCI-H460, RAW264.7, MM127, HepG2, MCF-7 cell lines	Anti-inflammatory, chemical profiling of BV, Cytotoxicity
(Borojeni et al., 2020) [325]	BV (*Apis m.*)	Lung cancer Cervical cancer breast cancer	In vitro; A549, HeLa, MDA-MB-231 cell lines	Apoptosis, cytotoxicity
(Kabakci et al., 2023) [158]	BV	breast cancer	In vitro; MCF-10A, MCF-7 cell lines	Apoptosis, cell cycle arrest, cytotoxicity
(Hwang et al., 2022) [36]	BV (*Apis m.*)	Lung cancer	In vitro; A549 cell line	Anti-inflammatory, cytotoxicity
(Chahla et al., 2024) [326]	BV (*Apis m. syriaca*)	Glioblastoma	In vitro; U87 cell line In vivo; Animal: mice	Cytotoxicity, anti-tumor, brain multiform
(Abass et al., 2025) [37]	BV (*Apis m.*)	Ehrlich ascites carcinoma	In vitro: EAC cell line In vivo; Animal: mice Xenograft	Anti-inflammatory Liver
(El-Bassion et al., 2016) [327]	BV (honeybee)	Lung Cancer Colon Cancer Cervical Cancer Prostate Cancer Larynx Cancer Rhabdomyosarcoma Hepatocellular Carcinoma Breast Cancer	In vitro; HeLa, A549, HCT116, PC3, HEP-2C, RDA, MCF-7, HepG2 cell lines In vivo; Animal: rats	Cytotoxicity
(Soukhtanloo et al., 2019) [328]	BV (*Apis m.*)	Colon Cancer	In vitro; HT-29, L929 cell lines	Apoptosis, Gene Expression-Pathway
(Alalawy et al., 2020) [329]	BV (*Apis m.*)	Cervical Cancer	In vitro; HeLa cell line Nanoparticle delivery of BV by chitosan coating	Apoptosis, cytotoxicity
(Frangieh et al., 2019) [174]	BV (*Apis m.*)	Breast Cancer	In vitro; 3T3, MCF-7 cell lines	Anti-bacterial, chemical profiling of BV, Anti-oxidant activity,
(El-Didamony, Amer et al., 2022) [330]	BV	Prostate cancer	In vitro; OEC, PC3 cell lines Delivery of BV	Apoptosis, cytotoxicity, cellular toxicity
(Duffy et al., 2020) [331]	BV (*Apis m.*) and melittin	Breast cancer	In vitro; MDA-MB-231, MCF-7, HDFa, HEK293FT, MCF-10A, MCF-12A, SKBR3 breast cancer, SUM149, SUM159, T-47D, ZR-75-1 cell lines	Membrane interactions of melittin, Gene Expression-Pathway
(Duarte et al., 2022) [332]	BV (*Apis m.*)	Breast cancer Colon Cancer	In vitro; HT-29, MCF-7 cell lines	Chemical profiling of BV, Synergy with 5-FU, cytotoxicity
(Sengul et al., 2024) [333]	BV	Lung cancer	In vitro; A549 cell line	Synergy with stem cells
(Lebel et al., 2021) [334]	BV and melittin	Glioblastoma	In vitro; Hs683, T98G, U737 cell lines	Apoptosis, Cytotoxicity, Characterization of BV, anti-tumor
(M. Sarhan et al., 2020) [268]	BV (*Apis m.*)	Liver cancer	In vitro; HUh7it-1 cell line	Anti-viral, gene expression
(A. G. Kamel et al., 2024) [335]	BV	Breast cancer Hepatocellular Carcinoma	In vitro; HepG2, MCF-7, HSF cell lines Nano delivery by chitosan	Gene Expression-Pathway, anti-tumor
(Amar et al., 2021) [336]	BV (*Apis m.*)	Tongue squamous cell carcinoma Melanoma	In vitro; TSCC, SCC25 cell lines	Synergy with cisplatin, Gene Expression-Pathway, cytotoxicity
(Shaimaa H. Shadeed, 2022) [337]	BV	Colon cancer	In vitro; Caco-2, HCT116 cell lines	Synergy with cetuximab, apoptosis, Gene Expression-Pathway, cytotoxicity
(Drigla et al., 2016) [338]	BV	Breast cancer	In vitro; MCF-7, HS578T cell lines	Synergy with propolis, Cytotoxicity
(Khamis et al., 2024) [339]	Not just BV, but also hesperidin and piperin	Breast cancer	In vitro; MCF-7 cell line In vivo; Animal: rats	Synergy with tamoxifen, Apoptosis, Gene Expression-Pathway, anti-angiogenesis
(Badivi et al., 2024) [340]	BV	Lung cancer	In vitro; A549 cell line Delivery of BV by PEGylate	Apoptosis, Gene Expression-Pathway, cytotoxicity
(Mirzavi et al., 2024) [56]	BV	Colon cancer	In vitro; C26 cell line In vivo; Animal: mice, Xenograft	Anti-inflammatory, Gene Expression-Pathway, Anti-oxidant activity, anti-tumor
(Babayeva et al., 2024) [341]	BV (*Apis m.*)	Hepatocellular Carcinoma Colon cancer Ewing sarcoma Prostate cancer	In vitro; HUH7, HT-29, Caco-2, A-673, SW-48, CARM-L12 TG3, PC-3 cell lines	Cytotoxicity
(Orman et al., 2025) [342]	BV (*Apis m.*)	Prostate cancer Breast cancer	In vitro; CCD34-Lu, HEK293 MDA-MB-231, PC3	Apoptosis, Delivery with mesoporous silica

Despite the increasing interest in BV as a possible anti-cancer treatment, there are still significant research gaps. Most investigations are confined to in vitro studies, with only a handful of in vivo models and no clinical trials so far, which impedes clinical application. There is a scarcity of detailed studies examining less prevalent cancers, mechanisms of drug resistance, long-term toxicity, and the comparative effects on healthy cells. Moreover, most research does not adequately address molecular mechanisms, immune modulation, or interactions within the tumor microenvironment. Although some advancements have been made with nanoparticle delivery systems, targeted and advanced delivery technologies still require further exploration. Inconsistencies regarding the source of the venom, its purification, and dosing also emphasize the need for standardization. The limited use of systems biology, bioinformatics, and personalized medicine underscores the need for stronger interdisciplinary research to fully realize bee venom’s therapeutic potential in cancer.

#### 4.3.2. Melittin in Cancer Research

In vitro cell culture has been widely implemented in cancer research regarding melittin as well as bee venom (Table 10). Preclinical studies using animal models, in particular mouse models, are also common regarding the anti-cancer effects of melittin (Table 11). Targeting various types of cancer, many studies involve xenografts (Table 11) and the inoculation of human cancer cell lines. Xenograft is the transplantation of living tissue from a different species or species to another. In cancer research, xenograft models are powerful preclinical tools created by transplanting human tumor cells into immunocompromised animals, typically mice. These models allow researchers to observe the growth, invasion, and metastasis of human cancer cells within a living organism. They are widely used to evaluate the efficacy and toxicity of newly developed anti-cancer agents, including chemotherapy, targeted therapies, and immunotherapies. Xenograft models employing aggressive human cancer cell lines such as MDA-MB-231 are particularly valuable for studying metastatic processes [113,117]. As a result, they provide critical insights during the preclinical stage, offering a more reliable foundation for the transition to human clinical trials (Table 10).

In many of the studies, a prominent experimental design choice is melittin analogs. These peptides are engineered—through design, hybridization, synthesis, and conjugation—to reduce melittin’s drawbacks, such as cytotoxicity and hemolysis, while enhancing stability against environmental degradation and improving therapeutic efficiency. Additionally, peptide design to target specific molecular interactions is a popular research topic [155,298,343,344] (Table 10). Components of these largely chemical interactions can be other peptides, therapeutic molecules, nanoparticles such as metal ions, or other biocompatible delivery elements [301,345,346]. For melittin synthesis, plasmid-based *E. coli* design is a noticeable method, alongside fungi. Not just as a stand-alone, melittin can be used as an enhancer for chemotherapy agents like cisplatin. In this case, membrane disruption potential plays a major role, and melittin may have synergistic effects or may behave like a delivery tool [200,331,347,348].

**Table 10:** In vitro cancer models for Melittin.

Article Name	Cancer Type	Cell Lines	Experimental Design	Additional Information
(Ebrahimdoust et al., 2023) [298]	Leukemia	Jurkat T, Raji cell lines	Melittin-derived cecropin-A-(CM11), Melittin hybrid	Apoptosis, Anti-tumor
(Sattayawat et al., 2025) [299]	lung cancer	Vero, A549, NCI-H460, NCI-H1975 cell lines	*Apis florea*, *Apis m.* Synergy with gefitinib	Apoptosis, Cytotoxicity Gene expression-pathway
(Alibeigi et al., 2025) [300]	Breast cancer	MCF-10A, SKBR3, and MCF-7 cell lines	*Apis cerana cerana* Melittin synthesis via *E. coli* Melittin-loaded pectin	Cytotoxicity, Gene expression-pathway, Hemolytic activity, Wound healing
(S. Liu et al., 2025) [346]	Cervical cancer	HeLa cell line	*Apis m.* Mel-7 Nanoparticle delivery by black phosphorous, Nanosheet	AMP, Anti-bacterial, Wound dressing/healing Cell viability
(Y. Li et al., 2025) [349]	Cervical cancer	HeLa cell line	Melittin analog design Melp5 analog Melittin-derived: d-m159	Apoptosis Gene expression-pathway Oxidative stress
(Zheng et al., 2024) [301]	Hepatocellular carcinoma, breast cancer	HepG2, 4T1, CT26cell line	Nanoparticle delivery of melittin: polydopamine	
(Hamze Mostafavi et al., 2025) [122]	Breast cancer	BT-474 NIH3T3	Delivery Synergy with Trastuzumab Melittin synthesis via *E. coli*	Immunomodulation
(Lischer et al., 2021) [350]	Breast cancer	MCF-7 cell line	Melittin purification	*Apis cerana*, Anti-tumor Cytotoxicity
(Sevin et al., 2023) [88]	Brain cancer	U87MG glioblastoma cell line	BV (*Apis m.*) Apamin PLA2	Cytotoxicity, Gene expression-pathway, Immunomodulation
(Yaacoub et al., 2022) [297]	Cervical Cancer	HeLa cell line	*Apis m.* PLA2	Anti-coagulation, Proteolytic activity, Cytotoxicity
(Zarrinnahad et al., 2018) [302]	Cervical Cancer	HeLa cell line	*Apis mellifera* Melittin purification	Apoptosis, Honey bee venom, Hemolytic activity Cytotoxicity
(Moghaddam et al., 2020) [351]	Breast cancer	4T1 cell line	Cisplatin Doxorubicin	Cytotoxicity Gene Expression-Pathway Hemolytic activity
(Bayat et al., 2022) [345]	Breast cancer	MCF-7 cell line	Delivery of melittin with nanoparticles Synergy	Cytotoxicity
(H. N. Lim et al., 2019) [306]	Melanoma	A375SM, SK-MEL-28, B16F10 cell lines	Not just melittin	Cell migration inhibition, Cell invasion, Gene expression-pathway
(Do et al., 2014) [247]	Skin cancer	SCC12, SCC25, NHK cell lines	*C. albicans*	AMP, Anti-fungal, Cytotoxicity, Skin diseases
(Y. Xiao et al., 2024) [343]	Breast cancer	MCF-7, SKBR3, MDA-MB-231 cell lines	Melittin derived; Mel-22, Mel-23a, Mel-23b Delivery of melittin	Stabilization of melittin Serum stability
(Daniluk et al., 2022) [352]	Breast cancer	MDA-MB-231, HFFF2, MCF-7 cell lines	Delivery of melittin with nanoparticles	Gene expression-pathway
(Dabbagh Moghaddam et al., 2021) [353]	Breast cancer	4T1 and SKBR3 cell lines	Delivery of melittin with nanoparticles: Niosomes	Gene expression-pathway Hemolytic activity Wound dressing/healing
(Jiang et al., 2019) [155]	Liver cancer	SMMC-7721 cell line	Melittin hybrid design Synergy with thanatin	AMP, Anti-bacterial, Hemolytic activity
(Y. Wu et al., 2017) [344]	Liver cancer	SMMC-7721 and HepG2 cell lines	Melittin derived; Mel-S4, Mel-S3, Mel-S1, Mel-S2	Hemolytic activity
(Sahsuvar et al., 2023) [159]	Cervical cancer Breast cancer	HeLa, 3T3, C33A, NSF, MCF-7 cell lines	Synergy Melittin hybrid Conjugate	Anti-bacterial, Anti-oxidant activity, Cytotoxicity, Folic acid, Hemolytic activity
(E. Han et al., 2023) [354]	Breast cancer	MCF-7 cell line	Delivery of melittin Not just melittin Doxorubicin	Gene expression-pathway Drug resistance Chemotherapy
(Jamasbi et al., 2018) [162]	Gastric cancer	MKN-7, MKN-74, NUGC-3 cell lines	Melittin synthesis via *E. coli*	Anti-bacterial, Cytotoxicity ROS, Hemolytic activity
(Kyung et al., 2018) [347]	Lung, Breast and Cervical cancer	A549, NCI-1299, MCF-7, HeLa cell lines	Delivery of melittin	Apoptosis Membrane interactions Cell penetrating Cytotoxicity
(M. Su et al., 2016) [355]	Ovarian cancer	SKOV3 cell line	Melittin-derived; atf-melittin Melittin synthesis via fungi	Anti-tumor Honey bee venom
(Qi et al., 2020) [356]	Cervical cancer	HeLa cell line	Delivery of melittin with nanoparticles	Apoptosis Cytotoxicity
(Honari et al., 2024) [357]	Non-small cell lung cancer	A549, Calu-3, MRC-5 cell lines	Delivery of melittin with nanoparticles: niosomes	Apoptosis Cytotoxicity Wound dressing/healing
(Ertilav & Nazıroğlu, 2023) [305]	Glioblastoma	DBTRG-05MG cell line	Cisplatin Synergy	Apoptosis Anti-tumor Cytotoxicity Gene Expression-Pathway Honey bee venom Oxidant activity
(Duffy et al., 2020) [331]	Triple negative breast cancer	SKBR3, MDA-MB-231, MCF-10A, HEK293FT, SUM149, SUM159, MCF-12A, HDFa, T-47D, ZR-75-1, MCF-7 cell lines	Delivery BV *Bombus terrestris*	*Apis m.* Membrane interactions of melittin Gene Expression-Pathway
(El-Didamony et al., 2024) [187]	Colon cancer Liver cancer	HCT116, Wi-38, Huh7 cell lines	Melittin-derived: melittin alcalase-hydrolusate Melittin hybrid Characterization of BV	Anti-bacterial Anti-biofilm Anti-tumor *Apis m.* Cell migration inhibition Cytotoxicity Multifunctional bioagent
(Zamani et al., 2024) [304]	Colorectal cancer	HCT116 cell line		Cytotoxicity Gene Expression-Pathway Autophagy
(Z. Jin et al., 2018) [358]	Bladder cancer	T24 and 5637 cell line		Gene expression-pathway
(Sangboonruang et al., 2020) [359]	Melanoma	NIH3T3 and A375 cell lines		*Apis florea* Apoptosis Gene Expression-Pathway Cytotoxicity
(H. Li, 2024) [360]	Lung cancer	H1299 and A549 cell lines		Gene expression-pathway Anti-angiogenesis Anti-tumor
(Kreinest et al., 2020) [361]	Hodgkin lymphoma	KM-H2 and L-428 cell lines	Cisplatin Synergy	Cytotoxicity Chemotherapy resistance
(Tipgomut et al., 2018) [362]	Human Bronchogenic Carcinoma Lung cancer	ChaGo-K1, THP-1 Wi-38 cell lines		Apoptosis Cell cycle arrest Cytotoxicity *Apis m.*
(X. Li et al., 2022) [363]	Lung cancer	A549 cell line		Apoptosis Ferroptosis Wound dressing/healing ROS
(Kong et al., 2016) [364]	Gastric cancer	SGC-7901 Cell line		Apoptosis ROS Gene Expression-Pathway
(Zorilă et al., 2020) [348]	Colon cancer Osteosarcoma Liver cancer	HT-29, MG-63, HepG2, L929 cell lines	Liposome In silico analysis	AMP Membrane interactions
(Q. Chen et al., 2019) [365]	Liver cancer	Huh7, SMMC-7721 and HepG2 cell lines		Gene expression-pathway
(J. Yao et al., 2020) [366]	Bladder cancer	5637 and UM-UC-3 cell lines		Anti-metastatic Cell migration inhibition Gene Expression-Pathway
(Mir Hassani et al., 2021) [367]	Breast cancer	MDA-MB-231 cell line		Anti-angiogenesis Anti-tumor Gene Expression-Pathway
(X. Wang et al., 2017) [303]	Pancreatic cancer	SW1990, Capan1, AsPC-1, BXPC-3 and HEK293T cell lines	Gemcitabine Synergy	Chemotherapy resistance Anti-tumor Gene Expression-Pathway
(X. Li et al., 2023) [368]	Lung cancer	A549 cell line		Anti-tumor, Autophagy Apoptosis, Gene Expression-Pathway
(Salimian et al., 2022) [369]	Breast cancer	MDA-MB-231 cell line		Anti-metastatic, Cell migration inhibition, Cytotoxicity, Gene Expression-Pathway
(Z. Zhang et al., 2016) [370]	Human hepatocellular carcinoma	Bel-7402, Hep3b, Huh7, HUVEC, HepG2, LO2, SMMC-7721, MHCC97-H cell lines		Anti-angiogenesis Anti-metastatic *Apis m.* Gene Expression-Pathway
(Jeong et al., 2014) [371]	Breast cancer	MDA-MB-231 and MCF-7 cell lines	*Apis m.*	Cell invasion Gene Expression-Pathway
(J.-Y. Huang et al., 2021) [372]	Gastric adenocarcinoma	AGS cell line		Anti-metastatic Gene Expression-Pathway Wound dressing/healing
(Y. Lv et al., 2022) [373]	Breast cancer Hepatocellular carcinoma	MCF-7, Hepa1-6 cell lines	Melittin analog Melittin synthesis In silico analysis *Apis m.*	Anti-tumor Cytotoxicity Hemolytic activity Molecular dynamics
(H. Jung et al., 2022) [312]	Cervical cancer	BEAS-2B, RAW264.7, RBL-2H3, HeLa cell lines	Melittin derived	Anti-inflammatory Allergy Anti-oxidant activity Cytotoxicity
(Plasay et al., 2022) [374]	Breast cancer	MCF-7 cell line		Apoptosis Gene Expression-Pathway
(Ceremuga et al., 2020) [375]	ALL, CML	CCRF-CEM, K-562 cell lines	*Apis m.*	Apoptosis
(Plasay & Muslimin, 2024) [376]	Colorectal cancer	WiDr cell line		Gene expression-pathway Cytotoxicity Honey bee venom
(Obeidat et al., 2023) [377]	Leukemia	K-562 cell line	BV Melittin purification	Apoptosis Cell cycle arrest *Apis m.* Cytotoxicity
(Alonezi et al., 2017) [378]	Ovarian cancer	A2780, A2780CR cell lines	Synergy with cisplatin Delivery of melittin	Activity/mechanism Cytotoxicity
(Lebel et al., 2021) [334]	Glioblastoma	Hs683, U737, T98G cell lines	Characterization of BV BV	Apoptosis Anti-tumor Cytotoxicity
Li et al., 2018 [307]	Lung cancer Cervical cancer	A549 and HeLa cell lines	Delivery by nanoparticles: zeolitic imidazolate	Gene expression-pathway
(Wattanakul et al., 2019) [379]	Colon cancer	Caco-2 cell line	Delivery of melittin with nanoparticles: alginate	Chemotherapy enhancement
(H. Lai et al., 2017) [380]	Breast cancer	MCF-7 Cell line	Delivery of melittin with nanoparticles: nanodiamonds	Cytotoxicity
(Nikodijević et al., 2024) [381]	Colon cancer	HT-29 and MRC-5 cell lines		Apoptosis Drug resistance Cytotoxicity
(M. C. Shin et al., 2016) [382]	Glioblastoma Cervical cancer	U87MG, LS174T, MDCK, CT26 and HeLa cell lines	Gelonin synergy Characterization Melittin Genetic design	Anti-tumor Cytotoxicity Ribosome inhibition
(Maani et al., 2023) [383]		In silico analysis	Melittin hybrid design In silico analysis	Molecular dynamics of melittin
(Keykanlu et al., 2016) [384]	Breast cancer	MCF-7 cell line	Delivery of melittin with nanoparticles: Perfluorooctyl Bromide Synergy with lactoferrin	Hemolytic activity
(S.-K. Zhang et al., 2016) [200]	Glioblastoma Cervical cancer	HeLa cell line	*Apis m.* Melittin-derived peptide: AR-23, RV-23	AMP Anti-bacterial Hemolytic activity Membrane interactions
(Keil et al., 2020) [55]	Lung cancer	Jurkat T lymphocytes, A549 cell lines	Melittin is only reference molecule	Anti-inflammatory Immunomodulation Asthma disease Endosomal escape
(Rajabnejad et al., 2018) [385]	Lung cancer	L929 and A549 cell lines	*Apis m.* Delivery of melittin with AS1411	Alpha helical peptide Cytotoxicity Hemolytic activity
(C. Zhou et al., 2020) [386]	Esophageal carcinoma	TE1 and Het-1a cell lines	Synergy	Apoptosis Anti-tumor Cell migration inhibition Cell cycle arrest Gene Expression-Pathway ROS
(Soliman et al., 2019) [387]	Gastric adenocarcinoma	COLO205, HCT-15, AGS cell lines	Melittin	Membrane Interactions Cytotoxicity
(Nakagawa et al., 2020) [388]	Melanoma	A375, A2058 cell lines		Allium sativum Melittin is the only reference Cytotoxicity Hemolytic activity
(Gao et al., 2024) [389]	Lung cancer	A549 cell line	Melittin hybrid: melittin-mil-2	Anti-tumor Gene Expression-Pathway
(Erkoc et al., 2022) [57]	Breast cancer	HUVEC, MDA-MB-231, HEK293T, RAW264.7 and HMEC-1 cell lines	BV BV elements melittin derived	Anti-inflammatory Honeybee Gene Expression-Pathway *Apis m.* Anti-tumor
(Delvaux & Rice, 2022) [390]	Liver cancer	HepG2 cell line	Melittin derived; melP5 Melittin synthesis Conjugate Delivery of melittin	Endosomal escape with melittin
(Yan et al., 2022) [391]	Bladder cancer	T24, EJ, BIU87 SV-HUC-1 cell lines	Delivery of melittin RNA	Apoptosis Gene Expression-Pathway Anti-tumor
(Daniluk et al., 2019) [392]	Breast cancer	MDA-MB-231 and MCF-7 cell lines	Delivery of melittin with nanoparticles: graphene	Apoptosis, ROS, Cytotoxicity, Membrane interactions
(R. Wang et al., 2022) [393]	Cancer	In silico analysis	AMP Melittin derived	Membrane interactions
(Hussein et al., 2023) [394]	Breast cancer	MDA-MB-231, MCF-7 cell lines	Delivery of melittin	Carnosine Synergy with olaparib
(Gasanoff et al., 2021) [124]	T cell leukemia	Jurkat T cell line	Docking	Membrane interactions Immunomodulation
(Q. Liu et al., 2025) [395]	Hepatocelular carcinoma	293T, A20, COC1, Hepa1-6, Hepg2, Huvec, U937	Melittin	Membrane interactions Peptide design Hemolytic activity
(Raveendran et al., 2020) [396]	Breast Cancer	MDA-MB-231 and MCF-7 cell lines	Delivery of melittin	Cytotoxicity
(R. Wu et al., 2025) [397]	Ovarian cancer	SKOV3 cell line	Melittin	Apoptosis Gene Expression-Pathway Cell cycle arrest
(Feng et al., 2020) [398]	Colon Cancer	CT26 cell line	Delivery of melittin Hydrogel	Membrane interactions
(Motiei et al., 2021) [399]	Breast Cancer	MDA-MB-231 cell line	Delivery of melittin with nanoparticles: chitosan	Apoptosis Nano peptide: LTX-315 Synergy with miRNA34a
(Ibrahim et al., 2025) [400]	Lung cancer	A549 cell line		Gene Expression-Pathway Synergy
(Bahreyni et al., 2023) [110]	Breast and cervical cancer, melanoma	4T1, B16F10, HeLa, MDA-MB-231 cell lines	Synergy Delivery	Anti-tumor Melittin derived Immunomodulation

**Table 11:** In vivo animal model cancer research with melittin (cell lines used for induction of in vivo cancer models).

Article Name	Cancer Type	Cell Lines	Experimental Design	Additional Information
(H. Wang et al., 2025) [111]	Breast cancer	4T1 cell line	Delivery of melittin Conjugate Melittin synthesis Xenograft	Cytotoxicity Anti-tumor Immunomodulation Promelittin
(Song et al., 2023) [112]	Cervical cancer Melanoma	HeLa, B16F10-OVA, DC2.4-Gal8-GFP cell lines	Vaccine D-melittin Drug delivery	Immunogenicity Immunomodulation Cell viability
(Rocha et al., 2022) [401]	Bone cancer Colorectal cancer	HT-29 cell line	Xenograft	*Apis m.* Anti-metastatic Cell viability
(S. Jia et al., 2025) [402]	Osteosarcoma	K7M2 cells and BMDCs	Animal: mice Melittin-derived peptide	AMP Hemolytic activity
(H. Zhang et al., 2025) [403]	Glioblastoma	Hs683 and T98G cell lines	Delivery of melittin with nanoparticles: liposome Xenograft Synergy with Resveratrol	Anti-tumor Hemolytic activity Gene Expression-Pathway Hemolytic activity
(F. Jia et al., 2021) [404]	Non-small cell lung carcinoma, Ovarian cancer	NCI-H358 and SKOV3 cell lines	Delivery of melittin Xenograft	Anti-tumor Cytotoxicity Hemolytic activity
(S. Lv et al., 2021) [405]	Breast cancer Lung cancer Colon carcinoma	A549, CT26, 3T3, MDA-MB-231 cell lines	Delivery of melittin with nanoparticles D-melittin	Anti-tumor Hemolytic activity
(Shir et al., 2011) [113]	Glioblastoma, Breast cancer, Vulval epidermoid carcinoma	A431, U138MG, U87MG, MDA-MB-231 cell lines	Delivery of melittin Xenograft	Gene Expression-Pathway Interactions Hemolytic activity Immunomodulation
(S. Kim et al., 2022) [406]	Breast cancer Acute Monocytic Leukemia	4T1 and THP-1 cell lines	Delivery of melittin Hybrid design Xenograft	Anti-metastatic Honeybee Hemolytic activity
(X. Kang et al., 2024) [169]	Hepatocellular carcinoma	HepG2 cell line	*E. coli*, *K. pneumoniae*, *S. aureus* Not just melittin	Anti-bacterial AMP Bacterial vaginosis disease Cytotoxicity
(Y. Wang et al., 2025) [407]	Lung cancer	A549 lung cancer cell line (A549/DDP)	Xenograft	Gene Expression-Pathway Chemotherapy resistance Honeybee
(J. Zhang et al., 2023) [408]	Hepatocellular carcinoma	BHK-21, L02, epG2 cell lines	Delivery of melittin with nanoparticles Xenograft	Cytotoxicity Membrane interactions Hemolytic activity
(X. Yu et al., 2019) [114]	Liver cancer, Colon carcinoma, Melanoma, Breast cancer	4T1, B16F10, CT26 cell lines	Delivery of melittin with nanoparticles Xenograft	Gene Expression-Pathway Anti-metastatic Anti-tumor Immunomodulation
(Chang et al., 2022) [409]	Breast cancer	MCF-7 and 4T1 cell lines	Melittin synergy with radiation Xenograft	Apoptosis *Apis m.* Anti-tumor
(P. Wu et al., 2022) [115]	Breast cancer, Hepatocellular carcinoma	4T1 and HEP1-6 cell lines	Not just melittin Delivery of melittin with siRNA nanoparticles Synergy Xenograft	Anti-metastatic Anti-tumor Cold tumor Immunomodulation Apoptosis Pathway interactions
(P. Xu et al., 2024) [410]	Glioblastoma	U251 cell line	Xenograft	Gene Expression-Pathway Cell cycle arrest Anti-metastatic Anti-tumor
(Meng et al., 2024) [411]	Hepatocellular carcinoma, Cervical cancer, Leukemia,	HeLa, Huh7, HEK293T, K-562, HEK293, HepG2, Hepa1-6 cell lines	Delivery of vector Melittin analog design: p5RHH	Transduction Transfection
(S.-F. Zhang & Chen, 2017) [412]	Lung cancer	A549 cell line	Xenograft	Gene Expression-Pathway Cell migration inhibition *Apis m.* Wound dressing/healing Anti-angiogenesis Anti-tumor
(S. Zhang et al., 2021) [413]	Lung cancer	A549 cell line	Xenograft	Apoptosis Gene Expression-Pathway Chemotherapy resistance Anti-tumor Glycolysis inhibition
(H. Zhu et al., 2021) [414]	Bone cancer	143 B cell line	Xenograft	Gene Expression-Pathway Anti-metastatic
(Qin et al., 2016) [415]	Bone cancer	UMR-106 cell line	Xenograft	Anti-tumor Anti-angiogenesis Gene Expression-Pathway
(Yan et al., 2023) [416]	Prostate Cancer	DU145, PC3 cell lines	Synergy with cisplatin Xenograft	Wound dressing/healing Gene Expression-Pathway Cell migration inhibition Anti-tumor Cisplatin sensitivity
(R. Yu et al., 2021) [417]	Lung cancer	A549 and H358 cell lines	Xenograft	Apoptosis Gene Expression-Pathway Cytotoxicity Cell migration inhibition
(C. Lee et al., 2017) [116]	Lung cancer Papillary adenocarcinoma	LCC, MLE12, and H441 cell lines	Xenograft	Gene Expression-Pathway Anti-tumor ROS Immunomodulation
(Luo et al., 2023) [418]	Colorectal cancer	HCT116, HT-29, SW-480, CCD 841 cell lines	Xenograft	Apoptosis Anti-tumor
(X. Wang et al., 2018) [419]	Pancreatic cancer	PANC-1, SW1990, HPDE, PATU8988, HS766T and BCPC3 cell lines	RNA Xenograft	Gene Expression-Pathway
(X. Yu et al., 2020) [420]	Melanoma	B16F10 and E0771 cell lines	Delivery of melittin with nanoparticles Xenograft	Anti-tumor
(M. Liu et al., 2016) [117]	Lung cancer Liver cancer Breast cancer Ovarian cancer	A549, SMMC-7721, MDA-MB-231, SKOV3 and CTLL-2 cell lines	Melittin fusion design Xenograft	Anti-tumor Cytotoxicity Immunomodulation
(Guo et al., 2023) [118]	Breast cancer	4T1 cell line	Delivery of melittin with nanoparticles: metal-phenol Xenograft	Anti-tumor Hemolytic activity
(Cheng & Xu, 2020) [421]	Breast cancer Colon cancer	MCF-7, HCT116 cell lines	Delivery of melittin with nanoparticles: Melittin synthesis design Xenograft	Redox sensitivity
(Q. Zhao et al., 2022) [422]	Anaplastic thyroid carcinoma	CAL-62 and C-643 cell lines	Synergy with apatinib Xenograft	Gene Expression-Pathway Pyroptosis Anti-tumor
(Y. Xie et al., 2023) [423]	Liver cancer	Huh7 and HEK293 cell lines	Delivery of melittin-derived peptide	
(I.-H. Han et al., 2022) [121]	Melanoma	B16F10 and THP-1 cell lines	Xenograft	Anti-tumor Gene Expression-Pathway Immunomodulation Wound dressing/healing
(Khorsand-Dehkordi & Doosti, 2024) [313]	Breast cancer	MCF-7, MCF-10A, RAW264.7 and 4T1 cell lines	Melittin synthesis via *E. coli* Xenograft	Anti-tumor apoptosis Gene Expression-Pathway Hemolytic activity
(Rahman et al., 2025) [424]	Ovarian cancer	HEK293, KGN, OVCAR-3, SKOV3 cell lines	Animal: mice Xenograft	Gene Expression-Pathway
(Sun et al., 2025) [425]	Ovarian cancer	SKOV3 cell lines	Animal: mice İnjection Xenograft	Gene Expression-Pathway
(Pedro et al., 2025) [426]	Osteosarcoma	MG-63, UMR-106, D-17 cell lines	3D cell culture	Cytotoxicity Cell migration inhibition
(X. Xie et al., 2022) [427]	Osteosarcoma	143 B cell line	Animal: mice Xenograft	Apoptosis Gene Expression-Pathway
(Y. Li et al., 2018) [307]	Cervical cancer Lung cancer	A549, HeLa, U14 cell lines	Animal: mice Xenograft	Gene Expression-Pathway Anti-tumor Hemolytic activity Nanodelivery with zeolitic imidazole
(D. Zhang et al., 2025) [126]	Breast cancer	4T1	Animal: mice Injection Delivery of melittin with nanoparticles: hyaluronic acid (HA) and metal (Fe)	Anti-tumor ROS Anti-oxidant activity
(Dai et al., 2025) [125]	Breast Cancer		Animal: mice Delivery of melittin	Gene Expression-Pathway
(Tang et al., 2022) [119]	Melittin	B16F10, B16, MB-49, MC38, MC38-OVA	Animal: mice Delivery with MnO2 Vaccine	Anti-tumor Cytotoxicity Immunomodulation
(K. Yang et al., 2023) [120]	Melittin	B16-luc, B16F10	Animal: mice Vaccine Delivery with hydrogel	Anti-tumor Cytotoxicity Hemolytic activity Immunomodulation
(Shen et al., 2024) [123]	Melittin	CT26, NIH3T3, HUVEC, CAF	Animal: mice Delivery Synergy	Hemolytic activity Immunomodulation

Although melittin has been more extensively studied in vivo than bee venom, several notable limitations on melittin research become apparent. Most in vitro studies focus on common cancers like breast, cervical, and lung, while aggressive types such as pancreatic, esophageal, and blood cancers remain underexplored. Research often relies on a few standard cell lines (e.g., HeLa, MCF-7, A549, 4T1), limiting model diversity. Although nanoparticle delivery systems and melittin analogs are being developed, variability in venom sources, delivery methods, and design strategies hinders standardization. In vivo studies are few, mainly in mouse xenografts, with little progress toward clinical trials. Moreover, while cytotoxicity and apoptosis are well studied, interactions with the tumor microenvironment, immune response, metastasis, and chemotherapy resistance are less understood. Additionally, multi-omics or systems are deficient biology approaches that could illuminate the comprehensive biological effects. Lastly, long-term animal studies inadequately address the toxicity and off-target consequences—particularly hemolytic activity. Closing these gaps is vital for advancing melittin from experimental treatment to clinical use in cancer care.

In light of the identified research gaps in both in vitro and in vivo investigations, future studies exploring melittin and bee venom for cancer treatment should focus on several important areas. To enhance the therapeutic significance of melittin, it is crucial to broaden the range of cancer models, particularly by including rare, treatment-resistant, and metastatic forms. Additionally, future research should aim to diversify the cell lines used and implement 3D cultures or organoid systems that more accurately represent the tumor microenvironment. In vivo investigations should advance beyond simplistic xenograft models to encompass metastatic and immunocompetent systems, allowing for a more accurate assessment of effectiveness, toxicity, and immune reactions. Standardized melittin analogs and delivery systems are needed to reduce hemolysis and improve tumor selectivity. Applying multi-omics and systems biology approaches may uncover mechanisms, resistance pathways, and biomarkers for therapy monitoring. Studies exploring the combination of melittin with current chemotherapeutics, immunotherapies, or radiotherapies should be expanded through well-planned synergy assessments, along with thorough long-term safety investigations. Ultimately, it will be essential to address the translational gap with comprehensive preclinical toxicology, pharmacokinetics, and clinical trial frameworks to propel melittin-based therapies toward practical oncology use.

## 5. Anti-Oxidant Activity

Anti-oxidant activity refers to the capacity of a substance to neutralize free radicals (harmful molecules such as Reactive Oxygen Species, ROS). Free radicals can cause oxidative stress in cells, damaging DNA, proteins, and lipids [162]. This contributes to the development of many diseases, such as aging, cancer, cardiovascular disease, and neurodegenerative disorders.

Bee venom and its main component, melittin, are not only notable for their anti-inflammatory, anti-microbial and anti-cancer effects, but also for their anti-oxidant activity (Table 12). Melittin has the potential to neutralize free radicals and reduce cellular damage associated with oxidative stress. Therefore, bee venom and melittin are considered protective or supportive agents in diseases where oxidative stress plays a key role.

## 6. Conclusions

**Figure 3:**
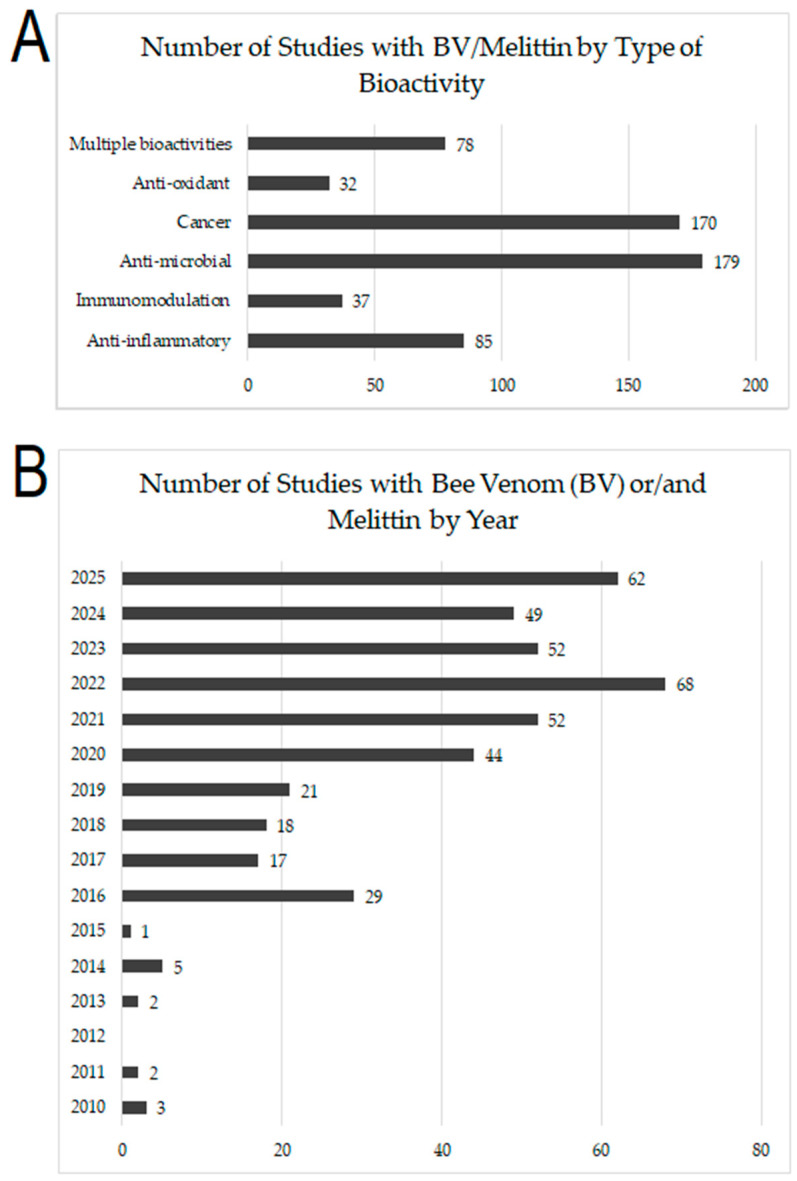
(**A**) The number of studies related to specific bee venom (BV)/melittin (M) bioactivity; (**B**) The overall number of relevant studies published between 2010–2025.

This scoping review aims to comprehensively update the current research on the therapeutic potential and bioactivity of bee venom (BV) and its major protein melittin (MEL), which could be systematically (meta)analyzed in future work. It highlights the research fields where BV, MEL and other compounds were predominantly studied while at the same time identifying research gaps and fields that have received comparatively less attention. Figure 3 shows the growing publication numbers on bee venom (BV) and melittin (M), particularly regarding their anti-microbial (overall 179 studies) and anti-cancer (overall 170 studies) effects, followed by anti-inflammatory (overall 85), immunomodulatory (overall 37), and anti-oxidant (overall 32) properties (Figure 3A). Publication trends reveal a sharp increase since 2020, peaking in 2022 (68 studies) and remaining high through 2025 (62 studies so far) (Figure 3B), highlighting sustained scientific attention to their therapeutic potential.

The literature is largely relevant to anti-cancer and anti-microbial activities. The main reason for that is that cancer and infectious diseases are among the major public health issues worldwide. Another reason is the availability of numerous in vitro protocols, thus making it relatively easy to conduct anti-cancer and anti-microbial studies. Anti-cancer research commonly emphasizes the detection of apoptosis-related markers in vitro, anti-metastatic effects, and synergy with conventional chemotherapeutic agents (Table 9 and Table 10). In vivo studies are less frequent and are predominantly conducted using pure melittin rather than bee venom (Table 11). *E. coli*, *S. aureus*, and *C. albicans* are the most studied micro-organisms, and drug/antibiotic resistance is the most popular area for BV/melittin anti-microbial research (Table 8). After the COVID-19 pandemic in 2020, not surprisingly, studies on the effects of BV/melittin against SARS-CoV-2 and MERS viruses have increased.

The anti-inflammatory effects of BV and MEL on rheumatoid arthritis have been extensively studied using animal models (Table 3, Table 4 and Table 5). However, research on their effects on Parkinson’s and Alzheimer’s diseases remains limited. Although these neurodegenerative disorders are associated with inflammation, the required experimental models are technically challenging and relatively costly. Inflammatory conditions are closely linked to immunological deficiencies, which need to be addressed through immunomodulatory approaches. The immunomodulatory activity of BV/MEL has been relatively less studied (Table 6), and most of the existing research focuses on the effects of MEL in cancer models (Table 7). This suggests that anti-cancer studies involving BV/MEL extend beyond cancer treatment alone, also contributing to our understanding of their immunomodulatory potential.

Studies on anti-oxidant activity of BV and/or melittin include a broad spectrum of in vivo (ducks, mice, rats, fish, quail) and in vitro (cell lines such as MCF-7, HeLa, Raw264.7, HT22, and others) models, targeting diseases including neurodegenerative and inflammatory diseases, cancer, diabetes, and colitis. Many studies report anti-oxidant effects alongside anti-inflammatory, cytotoxic, and immunomodulatory, often through gene expression analysis, DPPH assays, or in synergy with other agents (e.g., L-DOPA, cordycepin, propolis, Cu^2+^) (Table 12). BV is delivered through various systems, including injection, microneedles, nanoparticles, and acupuncture, reflecting growing interest in targeted or alternative therapies. Additionally, multiple studies investigated the chemical profile and hemolytic activity of BV, underlining its complex bioactivity and potential for both therapeutic interventions and toxicity.

Clinically, BV and MEL show promise as adjunctive or alternative therapeutic agents, particularly in conditions where conventional treatments are limited. Their strong anti-bacterial and anti-inflammatory activities point to possible applications in wound healing, drug-resistant infections, and chronic inflammatory diseases. In oncology, MEL pro-apoptotic and cytotoxic properties can be used in targeted delivery systems to minimize systemic toxicity while treating aggressive or resistant tumors. Melittin appears more advantageous in clinical trials than bee venom, as it is a peptide, suggesting that it can be produced synthetically under strict laboratory conditions without the need for beekeeping. On the other hand, bee venom is a mixture of numerous compounds that might act synergistically.

In the current body of research, in vivo studies are predominantly related to investigations of anti-inflammatory, anti-oxidant, and immunomodulatory effects implementing organism-level models necessary to reveal systemic immune interactions across multiple tissues and organs (Table 13). In contrast, cancer research relies largely on in vitro approaches, implementing well-characterized cell lines, the relative simplicity of modeling single-tissue pathologies, and reduced infrastructure requirements. Nevertheless, clinical studies remain limited due to ethical, logistical, and technical constraints. Anti-microbial investigations may be conducted either in vitro or in vivo, because microorganisms are simple unicellular organisms (Table 13). Furthermore, melittin is more frequently investigated in cancer-related studies than bee venom (BV), given that BV composition varies depending on species and collection methods, whereas melittin, as its principal component, is commercially available in standardized form of a well-defined chemical structure.

Conclusively, bee venom and its major compound, melittin, are among the most promising natural compounds due to their diverse biological activities. Their well-documented anti-inflammatory, anti-microbial, immunomodulatory, anti-cancer, and anti-oxidant properties suggest potential applications in the prevention and treatment of various diseases. Melittin’s ability to modulate cellular signaling pathways to suppress inflammation, regulate immune responses, combat microbial agents, reduce oxidative stress, and inhibit the proliferation and spread of cancer cells highlights its significant pharmacological value. These multifaceted effects underscore the need for continued research on bee venom and melittin, as well as further evaluation in clinical trials.

## Figures and Tables

**Table 1:** Major compounds detected in bee venom [4,6,11,15].

Type of Bee Venom Elements	Bee Venom Elements
Enzymes	Phospholipase A2 (PLA2), phospholipase B (PLB), hyaluronidase, phosphatase, and α-glucosidase
Peptides	Melittin, apamin, mast cell degranulating (MCD) peptide, adolapin, tertiapin, secapin, melittin F, cardiopep, procamine, minimine
Other molecules	Phospholipids, histamine, dopamine, noradrenaline, c-aminobutyric acid, a-amino acids, glucose, fructose, pheromones, isopentyl acetate, isopentanol, n-butyl acetate, n-hexyl acetate, 2-nonanol, n-octyl acetate, n-decyl acetate, benzyl alcohol, benzyl acetate, Ca, Mg, P, and lipids

**Table 2:** The search terms for the databases.

Stand-Alone Search Terms	Additional Terms for Combinations	
	General Terms	Suggested Terms
**Bee Venom**	Anti-bacterial, anti-fungal, anti-microbial, anti-viral, bioactivity, anti-cancer, phospholipase, therapy	Biofilm, fungus, hyaluronidase
**Melittin**	Anti-bacterial, anti-fungal, anti-microbial, anti-viral, bioactivity, cancer	Cell, immunogenicity, signal

**Table 3:** Anti-inflammatory bioactivity exerted by BV/MEL on targeted diseases/disorders.

Article	Substance and Source	Disease	Experimental Design	Additional Information
(Ahmedy et al., 2020) [17]	Melittin (*Apis m.*)	Acetic acid-induced ulcerative colitis	Animal: mice Injection	Anti-oxidant activity Chronic disease Gene Expression-Pathway
(Y. M. Lee et al., 2020) [18]	Apamin (*Apis m.*)	Gouty Arthritis	RAW264.7 cell line, mouse macrophage-like cell	Gene Expression-Pathway
(Z. Li et al., 2025) [19]	Melittin (bee)	Heat stress-induced İmmune organ damage	Animal: duck Feeding	Gene Expression-Pathway
(Abd El-Hameed et al., 2021) [20]	BV (*Apis m.*)	Epilepsy	Acupuncture Animal: rats	Neurological
(Zahran et al., 2021) [21]	BV (*Apis m.*)	Cardiac dysfunction due to type 2 diabetes	Animal: rats Injection	
(Abdelrahaman et al., 2025) [22]	BV (*Apis m.*)	Gentamicin-induced kidney injury	Animal: rats Injection	Anti-oxidant Activity Gene Expression-Pathway Lipid peroxidation
(Aly et al., 2023) [23]	BV (*Apis m.*)	Epilepsy	Acupuncture Animal: rats	Anti-oxidant activity Neurological
(Gu et al., 2022) [24]	BV (*Apis m.*) Melittin	Acne vulgaris	Animal: rats Injection, SZ95 cell line Bacteria: *Cutibacterium* sp.	Inactivation of Akt/mTOR/SREBP Signaling Pathway
(Goo et al., 2021) [25]	BV (bee)	Gouty Arthritis	Acupuncture Animal model: rats	
(Badawi et al., 2020) [26]	BV (*Apis m.*)	Parkinson’s disease	Animal: mice Injection	Anti-oxidant activity Neurological Synergy with L-dopa
(H. Kim et al., 2020) [27]	Melittin (bee)	Cisplatin-induced acute kidney injury	Animal model: mice	Gene Expression-Pathway Immunomodulation
(D. Shin et al., 2018) [28]	PLA2 (bee)	Atopic dermatitis	Animal: mice Topical administration	Gene Expression-Pathway Interactions
(G.-H. Kang et al., 2020) [29]	PLA2 (bee)	Atheroclerosis	Animal: mice	Immunomodulation
(Baek et al., 2020) [30]	PLA2 (*Apis m.*)	Alzheimer’s disease	Animal: mice	Apoptosis Neurological Immunomodulation
(D.-W. Kang et al., 2021) [31]	BV (*Apis m.*)	Burn injury, pain	Animal: mice Injection	Anti-nociception Neurological
(Pinto et al., 2024) [32]	BV (*Apis m.*)	Breast, lung, gastric adenocarcinoma, cervical and colon cancer	HeLa, Caco-2, AGS, NCI-H460, PLP2, MCF-7, HaCaT, HFF-1 cell lines	BV production Delivery of BV with nanoparticles: niosome
(Danesh-Seta et al., 2021) [33]	Apamin (Apis)	Multiple sclerosis	Animal: mice	Neurological
(W.-H. Kim et al., 2017) [34]	Melittin (*Apis m.*)	Atopic dermatitis	Animal: mice HaCaT cell line	Gene Expression-Pathway Adverse Effects
(Shaik et al., 2023) [35]	Melittin (honeybee)	Diabetes mellitus	Animal: rats Injection	Anti-oxidant activity Synergy with Cordycepin Pro-angiogenetic Delivery with a nanoparticle
(Hwang et al., 2022) [36]	BV (*Apis m.*)	Lung Cancer	A549 cell line	Sweet bee venom
(Abass et al., 2025) [37]	BV (*Apis m.*)	Ehrlich ascites carcinoma	EAC cell line Animal: mice Xenograft	Liver
(Hegazi et al., 2023) [38]	BV (bee)	Chronic Neck Pain	Acupuncture Clinical Study	Adverse effects Anti-oxidant activity Hemolytic activity
(Yaghoubi et al., 2022) [39]	Melittin (*Apis m.*)	Ulcerative colitis	Animal: mice Feeding	Anti-oxidant activity Melittin synthesis via fungi
(Ghorbani et al., 2022) [40]	Melittin (bee)	Cerebellar ataxia	Animal: rats Injection	Anti-apoptotic Neurological
(Zan et al., 2024) [41]	Melittin (bee)	Sepsis-induced acute kidney injury	Animal: mice Injection, Hk-2 cell line	Gene Expression-Pathway Anti-cell death Ferroptosis
(T. Wang et al., 2016) [42]	Melittin (honeybee)	Myocarditis	Animal: mice Injection	Pathway interactions
(Leem, et al., 2021) [43]	Melittin (bee)	Acute kidney injury	Animal: mice Injection	Anti-apoptotic Anti-oxidant activity
(Fan et al., 2021) [44]	Melittin (bee)	Acute liver failure	Animal: mice Injection	Pathway interactions
(Aghighi et al., 2022) [45]	Melittin (honeybee)	Cerebellar ataxia	Animal: rats Injection	Anti-cell death Autophagy Neurological
(Vu et al., 2021) [46]	Melittin (*Apis m.*)	Intracranial arterial dolichoectasia	Animal: mice	Immunomodulation Hemolytic activity Delivery of melittin with nanoparticles: iron oxide
(X. Yao et al., 2024) [47]	Melittin (honeybee)	Ischemic stroke	Animal: rats Injection	Apoptosis Gene Expression -Pathway Neurological
(H. Kim et al., 2022) [48]	Melittin (bee)	Lumbar spinal stenosis	Animal: rats Injection	Gene Expression-Pathway Immunomodulation
(Z. Liu et al., 2023) [49]	Melittin (bee)	Allergic contact dermatitis	Animal: mice Injection	Allergy Pathway interactions Delivery of Melittin with nanoparticles
(Xing et al., 2024) [50]	Melittin (*Apis m.*)	Cerebral ischemia	Animal: mice Injection BV2 cell line	Gene Expression-Pathway Neuroprotection
(Nguyen, Yoo, An et al., 2022) [51]	BV (honeybee)	Scopolamine-induced neurodegeneration	Animal: mice Injection	Anti-oxidant activity Neuroprotection Pathway interactions Delivery of BV: microneedle
(Lee et al., 2020) [52]	BV (*Apis m.*)	Acute kidney injury	Animal: mice Injection	Anti-oxidant activity Anti-apoptotic
(J.-Y. Kim, Jang et al., 2021) [53]	PLA2 (bee)	Cholestatic liver disease	Animal: mice Injection	
(D. Shin et al., 2016) [54]	PLA2 (bee)	Acute lung inflammation Side Effects of Radiotherapy	Animal: mice Injection	Immunomodulation
(Keil et al., 2020) [55]	Melittin (bee)	Asthma	A549, Jurkat T cell lines GATA3 gene inactivation via siRNA	Immunomodulation
(Mirzavi et al., 2024) [56]	BV (honeybee)	Colon cancer	Animal: mice Injection C26 cell line Xenograft	Gene Expression-Pathway Anti-tumor Anti-oxidant activity
(Erkoc et al., 2022) [57]	BV (*Apis m.*) Melittin-derived	Breast cancer	HEK293T, MDA-MB-231 and RAW264.7 cell lines	Anti-tumor Pathway interactions
(H.-J. An et al., 2016) [58]	Melittin (*Apis m.*)	Renal fibrosis	Animal: mice Injection NRK-49F cell line	Anti-fibrotic Gene Expression-Pathway Kidney
(W.-R. Lee et al., 2014) [59]	Melittin (*Apis m.*)	Acne vulgaris	Animal: mice Injection HaCat cell line	Gene Expression-Pathway Skin disease Protective effects
(Bae et al., 2022) [60]	BV (bee) Melittin	Skin infection	Animal: mice Topical administration	Skin disease
(H. An et al., 2018) [61]	BV (*Apis m.*) Melittin	Atopic dermatitis	Animal: mice Topical administration HaCat cell line	Gene Expression-Pathway Skin disease
(M. Choi et al., 2023) [62]	Melittin (honeybee)	Alzheimer’s disease	Animal: mice Injection C166, RAW264.7 cell lines	Neurological Conjugate with iron oxide Hemolytic activity
(Z. Wang et al., 2025) [63]	Melittin	Colitis-Associated Mental Disorders	Animal: mice Delivery	Anti-depressant
(J. Yao, Chen et al., 2025) [64]	Melittin (Honeybee)	Osteoarthritis	Animal, Delivery	Hydrogel
(Nam et al., 2025) [65]	Apamin	Cerebellar ataxia	Docking	Gene Expression-Pathway Neurological
(Romanenko et al., 2025) [66]	BV	Apical periodontitis	Animal: rats Injection	Anti-inflammatory
(Ayoub et al., 2025) [67]	Apis m.	Hyperalgesia	Animal: mice	Gene Expression-Pathway Neurological, Pain
(J.-W. Yu & Lu, 2025) [68]	Melittin	Pulmonary fibrosis	Animal: mice	Gene Expression-Pathway Anti-fibrotic
(T. Yu et al., 2025) [69]	Melittin	Parkinson’s disease	HT22 cell line	Gene Expression-Pathway Neurological, Anti apoptotic
(Izbicka & Streeper, 2025) [70]	PLA2	Chronic disease		Hemolytic activity Anti-nociception
(M. Chen et al., 2024) [71]		Parkinson	Animal: mice SH-SY5Y cell line	Apoptosis Neurodegeneration
(Cho et al., 2025) [72]	BV	SLE nephritis	Animal: mice injection	Gene Expression-Pathway Skin diseases Immunomodulation
(Zeng et al., 2025) [73]	Melittin	Psoriasis vulgaris	Animal: mice HaCat, HUVEC, RAW264.7 cell lines	Topical administration Skin diseases
(Niu et al., 2024) [74]	Melittin	Periprosthetic osteolysis	Animal: rats	Gene Expression-Pathway

**Table 4:** Anti-inflammatory therapeutic research targeting rheumatoid arthritis.

Article Name	Substance	Experimental Design	Additional Information
(Yousefpoor et al., 2022) [76]	BV (*Apis mellifera*)	Animal: rats Topical administration	Delivery of BV with nanoparticles: nanoemulsion
(G.-M. Choi et al., 2021) [77]	PLA2 (honeybee)	Animal: mice Injection	Gene Expression-Pathway Immunomodulation
(L. Yang et al., 2023) [75]	Melittin (bee)	In silico analysis	Docking, Pharmacology
(S. E. Sharaf et al., 2022) [78]	BV (bee)	Acupuncture Clinical Survey	Other bee products
(F. Liu et al., 2023) [79]	Melittin (*Apis m.*)	Animal: mice Injection	Gene Expression-Pathway
(Choe & Kim, 2017) [80]	Melittin (*Apis m.*)	Animal: mice RAW264.7 cell line	Gene Expression-Pathway Immunomodulation
(Du et al., 2021) [81]	Melittin (bee)	Animal: mice, rats Injection	Delivery of melittin with hyaluronic acid via microneedle
(L. Jin et al., 2025) [82]	Melittin (bee)	Animal: rats Injection	Microneedle delivery of melittin
(Xiong et al., 2025) [83]	Melittin (bee)	Animal: rat, pig Acupuncture	Delivery of melittin by liposome microneedle

**Table 5:** Anti-inflammatory bioactivity of BV and melittin without a specific disease target.

Article	Substance	Experimental Design	Additional Information
(Praphawilai et al., 2024) [84]	BV (*Apis m.*)	Vero and RAW264.7 cell lines HSV-1, HSV-2 Virus, Nitric Oxide Reduction Assay	Anti-viral, Cytotoxicity Gene expression-Pathway
(W.-H. Kim et al., 2018) [85]	Melittin (*Apis m.*)	HaCaT cell line Bacteria: *Porphyromonas* sp.	Gene Expression-Pathway, Cytotoxicity
(Im et al., 2016) [86]	BV (honeybee)	BV2 cell line	Cytotoxicity Pathway interactions Neurological
(Malan et al., 2016) [87]	Melittin	RAW264.7 cell line	Anti-endotoxin Melittin is the only reference Polimiksin B
(Sevin et al., 2023) [88]	Not just melittin, also Apamin, Melittin, PLA2	U87MG and RAW264.7 cell lines	Immunomodulation
(Abu-Zeid et al., 2021) [89]	BV (*Apis m.*)	Animal: rats Injection	Anti-oxidant activity Neuroprotective effects
(H.-S. Lee et al., 2021) [90]	BV	MCF-10A and RAW264.7 cell lines	Allergy
(Streeper & Izbicka, 2022) [91]	PLA2 (*Apis cerana*)	Human erythrocytes	Venom immunotherapy
(Abbasi et al., 2023) [92]	BV (honeybee)	Animal: mice Injection	Gene Expression-Pathway
(Alqarni et al., 2018) [93]	Melittin (honeybee)	THP-1 cell line	Vaccine
(Eid et al., 2022) [94]	Melittin (*Apis m.*)	Animal: rats	Pathway interactions Synergy with Diclofenac
(Senturk et al., 2022) [95]	BV (*Apis m.*)	Animal: rats Injection	Anti-oxidant effects Liver, Skeletal Muscle Oxidative stress
(Jo et al., 2021) [96]	PLA2 (bee)	Peripheral blood mononuclear cells	Immunomodulation
(Tseng et al., 2025) [97]	Melittin	RAW264.7 cell line Bacteria: Bacillus subtilis	GAL1–MELT fusion protein synthesis via *E. coli* Anti-inflammatory activity of recombinant melittin
(Rășinar et al., 2025) [98]	BV (*Apis m.*)	2,2-Diphenyl-1-Picrylhydrazyl Assay	Chemical synthesis of melittin Anti-oxidant activity
(Q. Zhang et al., 2025) [99]	Melittin	Schwann cell lines	Gene Expression-Pathway Neurological
(H. Zhao et al., 2025) [100]	BV, melittin	Animal: mice	Skincare, aging
(Lomeli-Lepe et al., 2025) [101]	BV	Acupoint injection Animal: mice	Anti-oxidant activity Neuroprotection

**Table 6:** Immunomodulatory activity research articles (without cancer).

Article	Substance	Disease	Experimental Design	Additional Information
(Basuini, 2024) [102]	BV (*Apis m.*)		Animal: *Liza ramada* Feeding	Anti-oxidant activity Characterization of BV
(G.-M. Choi et al., 2021) [77]	PLA2 (honeybee)	Rheumatoid arthritis	Animal: mice Injection	Anti-inflammatory Gene Expression-Pathway
(G.-H. Kang et al., 2020) [29]	PLA2 (bee)	Atherosclerosis	Animal: mice Injection	Anti-inflammatory Gene Expression-Pathway
(Baek et al., 2020) [30]	PLA2 (*Apis m.*)	Alzheimer’s	Animal: mice Primary cell culture	Anti-inflammatory Apoptosis Neurological
(Alqarni et al., 2018) [93]	Melittin (honeybee)		THP-1 cell line Vaccine	Anti-inflammatory Gene Expression-Pathway Delivery of melittin
(Karimi et al., 2023) [103]	Melittin (honeybee)		Animal: mice Injection	
(H. Kim et al., 2022) [48]	Melittin (bee)	Lumbar spinal stenosis	Animal: rats Injection	Anti-inflammatory Gene Expression-Pathway
(Vu et al., 2021) [46]	Melittin (*Apis m.*)	Intracranial Arterial Dolichoectasia	Animal: mice Injection	Anti-inflammatory Hemolytic activity Delivery of melittin with nanoparticles: iron oxide
(Z. Liu et al., 2023) [49]	Melittin (bee)	Allergic contact dermatitis	Animal: mice Injection	Anti-inflammatory Gene Expression-Pathway Delivery with nanoparticles
(D. Shin et al., 2016) [54]	PLA2 (bee)	Acute lung inflammation	Animal: mice Injection	Anti-inflammatory Gene Expression-Pathway Side effects of chemotherapy
(Jo et al., 2021) [96]	PLA2 (bee)		PBMC cell line	Anti-inflammatory
(H. An et al., 2018) [61]	BV, Melittin (*Apis m.*)	Atopic dermatitis	HaCat cell line	Anti-inflammatory Gene Expression-Pathway Skin diseases
(Eweis et al., 2022) [104]	BV		FMDV (foot-and mouth disease virus)	Veterinary Immunogenicity
(Cho et al., 2025) [72]	BV	SLE nephritis Skin lesions	Animal: mice injection	Gene Expression-Pathway
(S. Kim, Kim et al., 2021) [105]	BV	Asthma	A549 cell line	Allergy Gene Expression-Pathway
(Seo et al., 2025) [106]	Melittin	Respiratory disease	Animal: mice	LNP-MEL Conjugate with mRNA
(Sylvestre et al., 2021) [107]	Melittin		Animal: mice	Delivery Conjugate with PEG L and D-melittin Immunogenicity

**Table 7:** Immunomodulatory activity exerted by BV and MEL in cancer research.

Article	Substance	Cell Lines	Experimental Design	Additional Information
(Bahreyni et al., 2023) [110]	Melittin	4T1, B16F10, HeLa, MDA-MB-231	Synergy Delivery	Anti-tumor Melittin derived
(H. Wang et al., 2025) [111]	Melittin	4T1	Animal: mice Xenograft	Anti-tumor Melittin synthesis Conjugate with Promelittin
(Song et al., 2023) [112]	Melittin	HeLa, B16F10-OVA, DC2.4-Gal8-GFP	Animal: mice Vaccine Drug delivery	Cell viability Immunogenicity D-melittin
(Sevin et al., 2023) [88]	BV (*Apis m.*), Apamin, Melittin, PLA2	U87MG		Cytotoxicity Gene expression-pathway
(Abass et al., 2025) [37]	BV (*Apis m.*)	EAC	Animal: mice Xenograft	Anti-inflammatory Liver
(Shir et al., 2011) [113]	Melittin	A431, MDA-MB-231, U138MG, U87MG	Animal: mice Delivery	Gene Expression-Pathway Hemolytic activity
(X. Yu et al., 2019) [114]	Melittin	4T1, B16F10, CT26	Animal: mice Delivery	Anti-metastatic Gene Expression-Pathway
(P. Wu et al., 2022) [115]	Melittin	4T1, HEP1-6	Animal: mice Synergy siRNA Delivery	Anti-metastatic melittin synthesis Apoptosis Gene expression-pathway
(C. Lee et al., 2017) [116]	Melittin	LCC, MLE12, H441	Animal: mice	Anti-tumor, ROS (reactive oxidative species) Gene expression-pathway
(M. Liu et al., 2016) [117]	Melittin	A549, CTLL-2, SMMC-7721, MDA-MB-231, SKOV3	Animal: mice Melittin fusion design	Anti-metastatic Cytotoxicity
(Guo et al., 2023) [118]	Melittin	4T1	Animal: mice Delivery	Anti-tumor Hemolytic activity
(Tang et al., 2022) [119]	Melittin	B16F10, B16, MB-49, MC38, MC38-OVA	Animal: mice Delivery with MnO2 Vaccine	Anti-tumor Cytotoxicity
(K. Yang et al., 2023) [120]	Melittin	B16-luc, B16F10	Animal: mice Vaccine Delivery with hydrogel	Anti-tumor Cytotoxicity Hemolytic activity
(Keil et al., 2020) [55]	Melittin	A549, Jurkat T	Delivery	Anti-inflammatory Melittin is only reference Endosomal escape with melittin Asthma
(I.-H. Han et al., 2022) [121]	Melittin	B16F10, THP-1	Animal: mice	Anti-tumor Gene expression-pathway
(Hamze Mostafavi et al., 2025) [122]	Melittin	BT-474, NIH3T3	Delivery Synergy with Trastuzumab	Melittin synthesis via Bacteria: *E. coli*
(Shen et al., 2024) [123]	Melittin	CT26, NIH3T3, HUVEC, CAF	Animal: mice Delivery Synergy	Hemolytic activity
(Gasanoff et al., 2021) [124]	Melittin	Jurkat T	Docking	Cytotoxicity Membrane interactions
(Dai et al., 2025) [125]	Melittin	Breast Cancer	Animal: mice Delivery	Gene Expression-Pathway
(D. Zhang et al., 2025) [126]	Melittin	4T1	Animal: mice Delivery Injection	Anti-oxidant activity With HA and Fe ROS

**Table 8:** Research articles describing the anti-bacterial (ABA), anti-fungal (AFA), and anti-viral (AVA) activity of bee venom and melittin.

Article	Exerted Bioactivity	Substance	Experimental Design	Additional Information
(W. Zhu et al., 2021) [128]	AMP, ABA	Melittin	*E. coli*	Melittin synthesis via *E. coli*
(Akbari et al., 2018) [129]	AMP, ABA	Melittin-derived peptides (MDP1, MDP2)	*S. aureus*, *E. coli*, and *P. aeruginosa*	Membrane damage
(Ludwig et al., 2025) [131]	AMP, ABA	Not just Melittin, but also cathelicidin-related AMP (CRAMP)		Melittin synthesis via *E. coli* Cathelicidin-related delivery Gene Expression-Pathway
(W.-H. Kim et al., 2018) [85]	ABA, cytotoxicity	BV (*Apis m.*), Melittin	*Porphyromonas gingivalis*	Anti-inflammatory Gene Expression-Pathway
(Enigk et al., 2020) [132]	AMP, ABA, AFA, hemolytic activity	Not just Melittin, but also nisin, lactoferrin, parasin-1 and LL-37	*C. albicans*, *P. aeruginosa*, *S. aureus*	
(Tanuğur-Samancı & Kekeçoğlu, 2021) [133]	ABA, AFA	BV (*Apis m.*)	*S. aureus*, *C. albicans*, *E. coli*	Anatolian BV content Melittin 40.57%
(J. Yang et al., 2017) [134]	ABA, Enzymatic, Hemolytic, Anti-fibrinolytic activity	*A. cerana* venom serine protease inhibitor (AcVSPI)	*Beauveria bassiana*, *Bacillus thuringiensis*, *E. coli*	Anti-microbial roles of AcVSPI
(Pérez-Delgado et al., 2023) [135]	ABA, Hemolytic and Anti-oxidant activity	BV (*Apis m.*)	*E. coli*, *P. aeruginosa*, *S. aureus*	
(Arteaga et al., 2019) [136]	ABA, Anti-biofilm	Apitoxin of *Apis m.*	16 *Salmonella* strains	
(Socarras et al., 2017) [137]	Anti-biofilm activity, ABA,	BV (*Apis m.*) and Melittin	Antibiotic-resistant *Borrelia burgdorferi*	Antibiotic resistance
(Y. Liu et al., 2023) [138]	AMP, ABA,	Melittin-derived peptide; Melittin-Thanatin fusion (MT-W)	Antibiotic resistant *E. coli*, *Streptococcus pyogenes*, and others	Comparison of melittin to MT-W
(Gourkhede et al., 2020) [139]	ABA, AMP, Hemolytic activity, Cytotoxicity	Melittin-derived peptide; Cecropin A (1–7)-Melittin (CAMA) and lactoferricin	Antibiotic resistant *Salmonella Enteritidis strains*, *E. coli*	Multi-drug resistance
(Shi et al., 2016) [140]	AMP, ABA	Melittin	*Xanthomonas oryzae*	Plant protection, Molecular effect of melittin on cell membranes, energy metabolism and nucleic acid & protein synthesis
(Huan et al., 2022) [141]	AMP, Hemolytic activity, cytotoxicity, ABA	Melittin derived; Mel-d1, and LVFF-CONH2	*E. coli*, *Listeria monocytogenes*, *Vibrio Parahemolyticus*	Comparison with melittin
(K. Bakhiet et al., 2022) [142]	ABA, AFA	BV (*Apis m.*)	*E. coli*, *S. aureus*, *Serratia marcescens*,	
(X. Su et al., 2023) [130]	ABA, cytotoxicity	Melittin	*E. coli*, *S. aureus*	Delivery of melittin, Therapeutic
(S. Han et al., 2016) [143]	ABA	BV (*Apis m.*)	Methicillin-Resistant *S. aureus* (MRSA)	Antibiotic resistance
(Dosler et al., 2016) [144]	AMP, ABA, anti-biofilm activity	BV (*Apis m.*), not just melittin	*E. coli*, *K. (Klebsiella) pneumoniae*, *P. aeruginosa*	Antibiotic resistance
(F. Yang et al., 2023) [145]	AMP, ABA, hemolytic activity	Not just melittin, cationic AMP	*S. aureus*, *E. coli*	Skin care
(Chudinova et al., 2016) [146]	AMP, ABA	Not just melittin, Warnerin	*E. coli*, *Staphylococcus epidermidis*	Delivery of melittin
(Lima et al., 2022) [147]	ABA, anti-biofilm, anti-adhesive activity	Melittin from BV	*Quinolone-resistant uropathogenic E. coli (UPEC)*	BV content analysis
(Mirzaei et al., 2023) [148]	ABA, cytotoxicity, and anti-biofilm activity	Melittin	MRSA *P. aeruginosa*	Synergy with antibiotics Gene Expression-Pathway Melittin synthesis
(Kuzmenkov et al., 2022) [149]	ABA	Apamin from BV (*Apis m.*)	MRSA, *E. coli*, *Enterococcus faecalis*	Pharmaceutical Simulation
(W. A. Sarhan & Azzazy, 2017) [150]	ABA, cytotoxicity	Not just BV, also flavonal		Nano delivery of bee venom
(Strömstedt et al., 2017) [151]	ABA, AFA	Not just BV (*Apis m.*), Cyclotides	*E. coli*, *S. aureus*, *P. aeruginosa*, *C. albicans*	BV derived peptides
(Zolfagharian et al., 2016) [152]	ABA	BV (*Apis m.*)	*E. coli*, *S. aureus*, *P. aeruginosa*,	Antibiotic resistance
(Gökmen et al., 2023) [153]	ABA	BV (*Apis m.*)	*E. coli*, *K. pneumoniae*, *S. aureus*	drug resistance, BV content analysis, MDR
(Moridi et al., 2020) [154]	AMP, ABA	Melittin	MRSA, *S. aureus*, *Serratia marcescens*	Melittin synthesis via *E. coli*
(Jiang et al., 2019) [155]	AMP, ABA, hemolytic activity, cytotoxicity,	Melittin-derived peptides; thanatin	*E. coli*, *Bacillus subtilis*, *Salmonella* Typhimurium	Synergy with melittin
(Lu et al., 2019) [156]	AMP, ABA	Melittin-derived peptide; Melittin-Graphene hybrid	*E. coli*, *S. aureus*	Nano Delivery Membrane interactions
(Akhzari et al., 2021) [157]	Anti-parasitic activity, Anti-inflammatory, cytotoxicity	Melittin	*Leishmania* sp.	Synergy, Gene Expression-Pathway
(Kabakci et al., 2023) [158]	ABA	BV (*Apis m.*)	*Aeromonas hydrophila*, *Lactococcus garvieae*, *Vibrio anguillarum*, *Yersinia ruckeri*	Gene Expression-Pathway, antibiotic resistance
(Sahsuvar et al., 2023) [159]	AMP, ABA, cytotoxicity, ROS, hemolytic, Anti-oxidant activity	Melittin-derived peptide; Melittin-folic acid	*E. coli*	Synergy
(Vaiwala et al., 2022) [160]	AMP, ABA	Melittin-derived; melittin-peptidoglycan	MRSA, *E. coli*, *S. aureus*	Membrane interactions
(H. Yang et al., 2024) [161]	AMP, ABA, ROS, anti-biofilm activity	Melittin	*E. coli*, *K. pneumoniae* *S. aureus*,	Anti-quorum sensing
(Jamasbi et al., 2018) [162]	ABA, ROS, Cytotoxicity, hemolytic activity	Melittin	*E. coli*, *K. pneumoniae*, *Acinetobacter baumannii*	Chemical synthesis of melittin
(Z. Li et al., 2023) [163]	ABA, Anti-oxidant activity	Melittin	Gut bacteria	
(Ravensdale et al., 2016) [164]	AMP, ABA, hemolytic activity	Not just melittin, mel12-26, bac8c peptides	*MRSA S. aureus*	Drug resistance
(Jeon et al., 2024) [165]	AMP, ABA, hemolytic, Anti-inflammatory Cytotoxicity Anti-biofilm activity	Not just melittin, Osmin	*K. pneumoniae*	Drug resistance
(Ji et al., 2017) [166]	AMP, ABA	Melittin derived; cecropin-A-melittin (CAM-W)	*Bacillus subtilis*, *E. coli*, *Streptococcus pyogenes*	Heterologous melittin synthesis in *Bacillus subtilis*
(F. Wang et al., 2023) [167]	AMP, ABA	Melittin-derived; melittin-EAP fibrils	*Bacillus subtilis*, *E. coli*, *Streptococcus pyogenes*, and others	Peptide synthesis Melittin fusion design
(Bevalian et al., 2021) [168]	AMP, ABA, cytotoxicity	Melittin	Vancomycin Resistance *S. aureus*	Antibiotic resistance Wound dressing
(X. Kang et al., 2024) [169]	AMP, ABA, cytotoxicity	Not just melittin, also Pexiganan, plectasin, and cathelicidin	*E. coli*, *K. pneumonia* *S. aureus*	Bacterial vaginosis disease
(El-Sayied Ali et al., 2024) [170]	ABA	BV	*Paenibacillus larvae*	Nano delivery of BV
(Vergis et al., 2021) [171]	AMP, ABA, cytotoxicity, hemolytic activity	Cecropin-A-melittin	*E. coli*, *Lactobacillus acidophilus*, *Lactobacillus rhamnosus*	Antibiotic resistance, Laboratory model *Galleria mellonella*
(L. Zhou et al., 2020) [172]	ABA	Melittin	*E. coli*, *Staphylococcus pasteuri*, *MET-GST* (Melittin and glutathione-S-transferase fusion)	Melittin synthesis via *E. coli*
(Abou Zekry et al., 2020) [173]	ABA, cytotoxicity	Not just BV, also other bee products	*E. coli*, *S. aureus*	Nano delivery of BV Synergy
(Frangieh et al., 2019) [174]	ABA, hemolytic, cytotoxicity, and Anti-oxidant activity	BV (*Apis m.* syriaca)	*E. coli*, *S. aureus*, *B. subtilis*	BV content analysis
(S. Xiao et al., 2019) [175]	AMP, ABA	Melittin	*E. coli*, *S. aureus*	Nano delivery Membrane interactions
(Bardbari et al., 2018) [176]	AMP, ABA, anti-biofilm activity	Not just melittin; melittin with imipenem and colistin	*A. baumannii*	Synergy, Gene Expression-Pathway
(Mirzaei et al., 2022) [177]	ABA	Melittin	*S. epidermidis*	Synergy with antibiotics
(Gong et al., 2023) [178]	AMP, ABA, hemolytic activity	Not just melittin	*E. coli*	Membrane interactions of melittin
(Stephani et al., 2024) [179]	AMP, ABA	Melittin	Gram-negative bacteria	Docking Membrane interactions
(Maiden et al., 2019) [180]	AMP ABA, Anti-inflammatory, Anti-biofilm activity	Not just melittin, Tobramycin	*P. aeruginosa*	Nano delivery Synergy
(Zarghami et al., 2022) [181]	AMP ABA, cytotoxicity	Melittin	MRSA	Nano delivery
(Birteksoz-Tan et al., 2019) [182]	AMP, ABA, Anti-biofilm activity	Melittin derived; Cecropin-A-melittin	*S. aureus*, *Legionella pneumophila*	
(Marques Pereira et al., 2020) [183]	AMP, ABA	Not just Melittin also BV (apitoxin)	MRSA	
(Liao et al., 2023) [184]	AMP, ABA	Not just Melittin, also G(IIKK)_3_I-NH_2_ (G_3_) and G(IIKK)_4_I-NH_2_ (G_4_) peptides	*E. coli*	
(Rouhi et al., 2024) [185]	ABA, Anti-biofilm activity	Melittin	*Listeria monocytogenes*	Gene Expression-Pathway
(Shams Khozani et al., 2019) [186]	AMP, ABA, Anti-biofilm activity	Melittin	*P. aeruginosa*	Melittin synthesis, MDR
(El-Didamony et al., 2024) [187]	AMP, ABA, cytotoxicity, Anti-biofilm activity	Melittin derived; melittin alcalase-hydrolusate	*E. coli*, *Bacillus cereus*, *Enterococcus faecalis*	BV content analysis
(Zarghami et al., 2021) [188]	AMP, ABA, cytotoxicity, Anti-biofilm activity	Melittin-derived; chitosan-antibiotic coating melittin	MRSA VRSA	Nano delivery by chitosan Synergy with antibiotic
(Galdiero et al., 2019) [189]	AMP, ABA, Anti-biofilm activity	Melittin	*K. pneumoniae*, *P. aeruginosa*, *Aeromonas caviae*	Drug resistance
(Alajmi et al., 2022) [190]	AMP, ABA	BV (*Apis m.* yemenitica, *Apis m.* carnica)	*E. coli*, *S. aureus*, *P. aeruginosa*, *Salmonella Typhimurium*	Antibiotic resistance
(Mandal & Mandal, 2024) [191]	AMP, ABA	Not just melittin; MM-GBSA and QM/MM	*Acinetobacter baumannii*	In silico analysis
(Saraswat, Wani et al., 2020) [192]	AMP, ABA, hemolytic activity, cytotoxicity	Melittin-derived; with ionic liquids	*E. coli*, *S. aureus*	Conjugate
(Chetty et al., 2022) [193]	ABA, hemolytic activity	Melittin-derived; cecropin-A-melittin analogs, CA(1–7)M(2–9)	*E. coli*, *P. aeruginosa*, *S. aureus*, *Bacillus subtilis*	
(L. Yu et al., 2021) [194]	ABA	Not just Melittin, also alpha helical peptide	MRSA, *Acinetobacter baumannii*	Comparison with melittin
(Rangel et al., 2020) [195]	ABA, anti-biofilm	Melittin	*Acinetobacter baumannii*	Membrane interactions
(AL-Ani et al., 2015) [196]	ABA, AFA	BV, melittin	*E. coli*, *Klebsiella* sp., *Staphylococcus* sp., *C. albicans*	Synergy with antibiotics Drug resistance
(Babaeekhou et al., 2023) [197]	ABA, anti-biofilm activity	Melittin	*Acinetobacter baumannii*	Synergy Gene Expression-Pathway
(Nehme et al., 2020) [198]	ABA	Melittin and PLA2 from BV (*Apis m.*)	*E. coli*,	Antibiotic resistance
(Zarghami, Ghorbani, Bagheri et al., 2021) [199]	ABA, Anti-inflammatory, anti-biofilm activity	Melittin	MRSA VRSA	Nano delivery by chitosan/bioactive glass/vancomycin coatings
(S.-K. Zhang et al., 2016) [200]	AMP, ABA,	Melittin derived; Ar-23 and rv-23	*E. coli*, *S. aureus*	Membrane interactions of melittin
(Pashaei et al., 2019) [201]	ABA	Melittin	Drug-resistant *Acinetobacter* spp.	Toxicity
(Brand & Khairalla, 2021) [202]	ABA	Melittin	Gram-negative bacteria	Membrane interactions
(Hakimi Alni et al., 2020) [203]	ABA, cytotoxicity Anti-biofilm activity	Not just melittin, also Mupirocin	MRSA MSSA	Synergy with melittin, Gene Expression-Pathway
(Pereira et al., 2023) [204]	ABA	Not just melittin, but also Oxacillin	MRSA	Synergy with melittin
(Mahmoudi et al., 2020) [205]	ABA, hemolytic activity,	Not just melittin, but also clindamycin	MRSA MSSA	Synergy with melittin, Gene Expression-Pathway
(Güven Gökmen et al., 2023) [206]	ABA, Anti-inflammatory	BV (*Apis m.*)	*Serratia marcescens*, *Acinetobacter lwoffii*, *Pseudomonas* sp.	Veterinary Antibiotic resistance, Subclinical mastitis (Cow disease)
(W.-R. Lee et al., 2014) [59]	ABA, Anti-inflammatory, cytotoxicity	Melittin	*Propionibacterium acnes*	Gene Expression-Pathway
(Rad et al., 2024) [207]	AMP, ABA, cytotoxicity, Hemolytic activity	Melittin-derived peptides; M1 and M2	*Staphylococcus* sp., *Enterococcus faecalis*, *E. coli*	
(Bae et al., 2022) [60]	ABA, Anti-inflammatory	Melittin and BV (*Apis m.*)	*Streptococcus pyogenes*	Animal Skin diseases
(Oehler et al., 2023) [208]	AMP, ABA	Melittin derived; Encapsulated	*E. faecium*, *S. aureus*, *A. baumannii*	Microemulsion (BMEs)
(M. Sharaf et al., 2024) [209]	Cytotoxicity, ABA, AFA	Apitoxin of BV (*Apis m.*) encapsulated in chitosan nanoparticles	*S. aureus*, *Staphylococcus hominis*, *E. coli*	Nanotreatment
(Ahmed et al., 2024) [210]	ABA, AFA	BV (*Apis m.*)	*E. coli*, *S. aureus*, *B. cereus*	Natural preservative
(M. Sharaf et al., 2023) [211]	Anti-biofilm activity, ABA	BV (*Apis m.*), Nanoflowers loaded BV (Bv-ZnO@αFe_2_O_3_)	54 fecal Antibiotic-resistant strains (*E. coli*, *Klebsiella* strains)	
(Ji et al., 2014) [212]	AMP, ABA, AFA	Melittin derived; Cecropin A–melittin mutants (CAM-W)	*E. coli*, *Campylobacter jejuni*, *Helicobacter pylori*	
(Gülmez et al., 2017) [213]	Cytotoxicity, ABA	BV (*Apis m.*)	Multi Drug Resistant (MDR) strains; *E. faecium*, *E. coli*	Gene expression pathway
(A. Kamel et al., 2021) [214]	Cytotoxicity, ABA	BV (*Apis m.*)	Multi Drug Resistant (MDR) 62 clinical bacteria isolates (*P. aeruginosa* strains)	Synergistic effect with antibacterial drugs
(Abdel-Monsef et al., 2023) [215]	Cytotoxicity, ABA, AFA	Superoxide dismutase (SOD) of BV (*Apis m.*)	*Proteus mirabilis*, *Salmonella typhi*, *C. albicans*	
(Sonmez et al., 2022) [10]	AMP, ABA	BV (*Apis m.*)	*S. aureus*, *B. cereus*, *S. enterica*	
(Sullivan et al., 2011) [216]	AMP, Anti-biofilm, ABA	Not just Melittin B, also C16G2, AMP G2	*Streptococcus mutans*	Mouth wash
(Maitip et al., 2021) [217]	AMP, ABA, AFA	BV (*Apis m.*, A. Cerana, A. Dorsata and A. Florea), melittin	*Staphylococcus* sp., *MRSA*, *Bacillus subtilis*	
(Tanuğur Samancı & Kekeçoğlu, 2022) [218]	Cytotoxicity, Anti-oxidant, anti-aging, ABA, AFA	BV, also bee products; honey, propolis, beeswax, and royal jelly.	*P. aeruginosa*, *S. aureus*, *E. coli*, *C. albicans*	Prototype body cream
(Radhakrishnan et al., 2024) [219]	AMP, Cytotoxicity, Hemolytic activity, anti-biofilm, ABA	Melittin-derived Mel-LX3	*MDR P. aeruginosa*, *MRSA*, *Staphylococcus* sp.	
(Celebi et al., 2023) [220]	Anti-biofilm, Cytotoxicity, ABA	BV	*E. coli*	Synergy of BV Combination with Amoxicillin-clavulanic acid
(Akbari et al., 2022) [221]	AMP, Cytotoxicity, Hemolytic activity, ABA	Melittin-derived peptides; MDP1,2	MDR strains of *S. aureus*, *E. coli*, and *P. aeruginosa*	De novo designed Melittin-derived peptides
(Harries et al., 2013) [222]	AMP, AFA	Not just Melittin, also PAF26, P113 and cecropin A peptides	*Saccharomyces cerevisiae* strains	Gene Expression-Pathway Membrane interactions
(Q. Chen et al., 2021) [223]	AMP, Hemolytic activity, Cytotoxicity, ABA	Melittin	*E. coli*, *Shigella flexneri*, *S. aureus*	Melittin synthesis via *E. coli*
(Ferreira et al., 2021) [224]	AMP, ABA	Melittin derived; Cecropin A-melittin hybrid peptide BP100, W-BP100	*P. aeruginosa*, *E. coli*, *S. aureus*, *Enterococcus faecalis*	
(Thankappan et al., 2023) [225]	AMP, Hemolytic activity, Cytotoxicity, ABA	Melittin and melnp	*P. aeruginosa*, *E. coli*, *S. aureus*	Melittin nanoparticles
(Askari et al., 2021) [226]	AMP, Hemolytic activity, Cytotoxicity, ABA	Melittin	Drug-resistant (XDR) *Acinetobacter baumannii*, MRSA, and *K. pneumoniae*	Melittin synthesis via *fungi*
(Elswaby et al., 2022) [227]	Anti-oxidant activity, ABA, AFA	BV and also propolis	*S.* Typhimurium, *E. coli*, *B. cereus*	
(Picoli et al., 2017) [228]	AMP, Anti-biofilm activity, ABA	Melittin	*S. aureus*, *E. coli* *P. aeruginosa*	
(Aburayan et al., 2022) [229]	AMP, Cytotoxicity, ABA, AFA	Melittin	MRSA, *P. aeruginosa*, *E. coli*	Delivery coated by Polyvinylpyrrolidone
(Alvarez et al., 2022) [230]	AMP, ABA	Melittin	Gram-negative and Gram-positive bacteria	Nano fiber design
(Hejníková et al., 2024) [231]	AMP, ABA	BV (*Apis m.*) and melittin	*E. coli*, *L. monocytogenes*	Gene expression analysis
(Choo et al., 2010)	AMP, ABA	Melittin, bombolitin	Gram-positive and two Gram-negative bacteria	
(Bui Thi Phuong et al., 2024) []	AMP, Hemolytic activity, Cytotoxicity, ABA, AFA	Melittin-derived peptides; BP52- based on Melittin M and Cecropin A	*B.s cereus*, *E. faecalis*, *L.monocytogenes*	
(S. Huang et al., 2024) [232]	AMP, Hemolytic activity, ABA	Melittin derived; Mel-C8	*S. aureus*, *E. faecalis*, *E. coli*	Membrane permeabilization
(Babaie et al., 2020) [233]	ABA	Not just BV (*Apis m.*). Also snake scorpion	*S. aureus*, *B. subtilis*, *P. aeruginosa*, *E. coli*	
(Pola et al., 2023) [234]	AMP, ABA, AFA	Other halictine peptides of BV	*A. baumannii*, *E. coli*, *S. epidermidis*, *S. aureus*	Delivery by polymer-PEP
(Nabizadeh et al., 2023) [235]	AMP, ABA	Melittin and Lasioglossin hybrid peptides	*A. baumannii* and *S. aureus*	Simulation study
(Chou et al., 2021) [236]	AMP, Anti-biofilm activity, Cytotoxicity, ABA, AFA	Not just melittin, also P19 peptide.	*E. coli*, *S. aureus*	Membrane permeabilization
(Saraswat, Aldahmash et al., 2020) [192]	AMP, Cytotoxicity, ABA	Not just Melittin, also combination with ionic liquids (ils)	*E. coli* and *S. aureus*	
(Pourahmadi et al., 2022) [237]	AMP, Anti-biofilm activity, ABA	BMAP27-Melittin conjugate	*39 Streptococcus* mutans strains	Clinical Isolates from Oral Cavity
(López-García et al., 2010) [238]	AMP, AFA	Not just melittin	*Saccharomyces cerevisiae*	Synergy Gene expression-pathway
(Y. Yang et al., 2020) [239]	AMP, Hemolytic activity, ROS activity, Cytotoxicity, AFA	Not just melittin, alfa helical peptide,	*C. albicans*	Drug resistance
(Tanuğur-Samanc & Kekeçoğlu, 2021) [133]	AFA, ABA	BV (*Apis m.*)	*C. albicans*, *E. coli* *S. aureus*	Chemical profiling of Anatolian BV
(Kočendová et al., 2019) [240]	AMP, AFA, Hemolytic, Cytotoxicity, anti-biofilm	BV-derived peptides	*Candida* strains	
(S.-B. Lee, 2016) [241]	AFA	BV (*Apis m.*)	*C. albicans*	
(El-Didamony, Kalaba et al., 2022) [242]	Anti-biofilm, AFA	BV	*C. albicans*, *Cryptococcus neoformans*, *Kodamaea ohmeri*	Delivery loaded on chitosan nanoparticles
(J.-Y. Kim, Park et al., 2020) [243]	AFA, AMP, ROS activity,	Melittin-derived Hn-Mc peptide	*Candida* sp., *Fusarium* sp., *Trichosporon* sp., *Aspergillus flavus*	Apoptosis
(J. Park et al., 2018) [244]	AFA	BV (*Apis m.*) and apamin	*Trichophyton rubrum*	
(Todorova et al., 2024) [245]	AFA, cytotoxicity, genotoxicity	BV	*Saccharomyces cerevisiae*	Gene Expression-Pathway, Oxidative stress
(Hilpert et al., 2023) [246]	AMP, AFA	Not just melittin, Cecropin A-melittin hybrid	*C. albicans*	BioSAXS measurements
(Do et al., 2014) [247]	AMP, AFA, cytotoxicity	Not just melittin, Cecropin A, protegrin-1 and histatin 5	*C. albicans*	Skin penetration
(Y.-M. Kim et al., 2024) [248]	AMP, AFA, ROS activity,	Melittin derived, WIK-14,	*C. albicans*, *Candida krusei*, *Candida parapsilosis*	Animal
(Mahmoud et al., 2024) [249]	AMP, AFA	BV (*Apis m.*)	*Vairimorpha ceranae*	Gene Expression-Pathway, Bee disease
(C. Park & Lee, 2010) [250]	AMP, AFA, ROS,	Melittin and BV (*Apis m.*)	*C. albicans*	Apoptosis
(E. J. Lim et al., 2022) [251]	AMP, AFA	Not just melittin, also magainin 2, cecropin A, and mastoparan B peptides	*C. albicans*, *Candida* sp.	Compared with melittin
(D. Yu et al., 2022) [252]	AMP, AFA, ROS, cytotoxicity, anti-biofilm activity	Melittin-derived peptides; lactoferrin and zinc loaded	*C. albicans*	Melittin nano delivery
(S.-H. Shin et al., 2017) [253]	AMP, AFA, cytotoxicity	BV, Melittin and Apamin	*Alternaria* and *Aspergillus* sp.	Gene Expression-Pathway
(G. N. Kim, Choi et al., 2021) [254]	Anti-viral peptide, AVA, cytotoxicity	Melittin-derived vaccine	*SARS-CoV-2*	Vaccine, Melittin signal peptide SARS-CoV-2
(Peskova et al., 2017) [255]	Anti-viral peptide, AVA, cytotoxicity, hemolytic activity,	Not just melittin, CAM-W, GALA, SMAP29, KALA peptides	*Lentivirus*	Ebola
(Praphawilai et al., 2024) [84]	AVA, cytotoxicity	BV (*Apis m.*)	*HSV-1*, *HSV-2*	Anti-inflammatory
(D.-H. Kim et al., 2020) [256]	AVA, cytotoxicity	BV	*HPV*	Apoptosis, Gene Expression-Pathway, SARS-CoV-2
(Männle et al., 2020) [257]	AVA	BV	*SARS-CoV-2*	SARS-CoV-2
(Hood et al., 2013) [258]	AVA, cytotoxicity,	Melittin delivery	*HIV-1*	Nano delivery
(E. Choi et al., 2016) [259]	AVA, cytotoxicity	Melittin delivery	*HIV-1*	Vaccine, immunogenicity
(Enayathullah et al., 2022) [260]	AVA, cytotoxicity	Not just melittin, also Gramicidin S	*SARS-CoV-2*	Therapeutic
(Dehghani et al., 2020) [261]	AVA	Melittin	*HIV*	In Silico Analysis, simulation
(Mustafa et al., 2023) [262]	AVA	BV elements	*Capripoxvirus*	In Silico Analysis
(Muzammal et al., 2022) [263]	AVA	BV elements; PLA2	*Ebola Virus*	In Silico Analysis
(Baldassi et al., 2022) [264]	AVA	Melittin	*SARS-CoV-2*	Membrane interactions Melittin nano delivery
(Uddin et al., 2016) [265]	AVA, cytotoxicity	Melittin and BV (*Apis m.*)	*Influenza A virus (PR8)*, *Vesicular Stomatitis Virus*, *Respiratory Syncytial Virus*, *Herpes Simplex Virus*	
(Chianese et al., 2023) [266]	Anti-microbial peptide, AVA, cytotoxicity	Melittin-derived peptides; RV-23 and AR-23	*Sandfly Fever Naples Virus (SFNV)*	
(Das Neves et al., 2016) [267]	AVA	Not just melittin	*Human immunodeficiency virus (HIV)*	Melittin nano delivery
(M. Sarhan et al., 2020) [268]	AVA, cytotoxicity	BV elements (Apamin, melittin, mast cell degranulating (MCD) peptide)	*Hepatitis C virus*	Gene expression
(Abd El Maksoud et al., 2024) [269]	AVA, cytotoxicity	Not just BV (*Apis m.*), also Vespa orientalis	*SARS-CoV-2*	In silico and In vitro
(Farhoudi et al., 2022) [270]	AVA	Melittin (*Apis m.*), melittin hybrid design	*SARS-CoV-2*	In silico analysis, docking
(Al-Rabia et al., 2021) [271]	AVA	Melittin and Angiotensin	*SARS-CoV-2*	In silico analysis, docking
(Elnosary et al., 2023) [272]	AVA, ABA	BV	*MERS-CoV*, *S. aureus*, *Bacillus subtilis*, *P. aeruginosa*	Delivery of bee venom loaded chitosan, nanoparticle
(Abd-El-Samie et al., 2024) [8]	AVA, Hemolytic activity, cytotoxicity	BV (*Apis m.*)	*Bee viruses; Black Queen Cell Virus* (*BQCV*), *Deformed Wing Virus (DWV)*, *Kakugo*, *Varroa Destructor Virus-1 (VDV-1)*	BV content analysis, Hyaluronidase and pla2
(Hartmann et al., 2016) [273]	AVA	Melittin	*Feline Immunodeficiency Virus (FIV)*	Cat study, Veterinary
(Z. Lai et al., 2024) [274]	AFA, ABA	Melittin	*E. faecalis*, *P. aeruginosa*, *Salmonella pullorum*	Peptide design
(Hu et al., 2025) [275]	ABA, AMP	BV	*E. coli* and *Salmonella enterica*	ROS, cytotoxicity Hemolytic activity
(Teiba et al., 2025) [276]	ABA	BV	*E. coli*	not just BV
(El-Bilawy et al., 2025) [277]	ABA	BV	*E. coli*, *E. faecalis*, *S.* Typhimurium	Chemical profiling of BV
(J. H. Kim et al., 2019) [278]	AFA	BV (*Apis m.*)	*Malassezia* spp. strains	Skin diseases
(C.-Y. Zhao et al., 2025) [279]	ABA	Melittin	*E. coli*	Melittin resistance
(Sonmez et al., 2025) [280]	ABA	BV (*Apis m.*)	*Paenibacillus larvae*	Chemical profiling
(Lima et al., 2021) [281]	ABA, Anti-biofilm	Melittin	MRSA, *S. aureus*	Wound dressing
(Fahad Alharbi et al., 2025) [282]	ABA, AMP, Anti-biofilm	Melittin derived, CMEL, CMEL-M1	*A. baumannii*	
(Kumar et al., 2025) [283]	ABA, AMP, Anti-biofilm	Melittin, 19 other AMPs (hirunipins)	*E. coli*, *S. aureus*, *S. epidermidis*	Melittin as positive control
(Ramos-Alcántara et al., 2025) [284]	ABA, AMP, Hydrolytic activity	Melittin	*E. coli*, *K. pneumoniae*	Gene Expression-Pathway
(Reyad et al., 2025) [285]	ABA	BV	*E. coli*, *K. pneumoniae*, *P. aeruginosa*	Synergy Chemical profiling Antibiotic resistance
(X. Xu et al., 2025) [286]	ABA	Melittin	*MRSA*, *E. coli*, *S. aureus*	Melittin-derived peptide synthesis
(X. Yang et al., 2025) [287]	ABA, hemolytic activity	Melittin	*S. pyogenes*	Glycolization of peptide,
(J. Yao, Li et al., 2025) [288]	ABA, AMP, Anti-oxidant activity	Melittin	Animal: rats *E. coli*, *S.aereus*	ROS, Delivery, Synergy with Cu^2+^
(X. Wu et al., 2016) [289]	ABA, AMP, Cytotoxicity	Melittin	Docking *Listeria* spp.	
(Ni et al., 2025) [290]	ABA, AMP, Proangiogenic activity	Melittin	Hydrogel	Delivery, Wound dressing
(Maleki et al., 2016) [291]	ABA, AMP	Melittin cecropin-A conjugate	*E. coli*	Nanodelivery with iron oxide
(Z. Yang et al., 2025) [292]	ABA, Anti- biofilm	Melittin	*S. aureus*, *P. aeruginosa Candida* sp.	ROS Food preservative
(Awad et al., 2025) [293]	ABA, Anti- biofilm	*Apis mellifera*	*Lactiplantibacillus plantarum*	Gene Expression-Pathway
(W. Chen et al., 2019) [294]	ABA, AMP, Cytotoxicity	Melittin	*E. coli*	Delivery, Membrane interactions
(Tseng et al., 2025) [97]	ABA, Anti-inflammatory	Melittin	*Bacillus subtilis*	GAL1–MELT fusion protein, Melittin synthesis via *E. coli*

**Table 12:** Research on the anti-oxidant activity of bee venom and melittin.

Article	Substance	Disease	Experimental Design	Additional Information
(Z. Li et al., 2025) [19]	Melittin	Heat stress induced immune organ damage in ducks	Animal: ducks	Gene expression Heat-stressed ducks
(Pérez-Delgado et al., 2023) [135]	BV (*Apis m.*)		Africanized honeybee	Anti-microbial Hemolytic activity
(Ahmedy et al., 2020) [17]	Melittin (*Apis m.*)	Acetic acid- induced ulcerative colitis	Animal: mice	Anti-inflammatory Chronic disease
(Rășinar et al., 2025) [98]	BV (*Apis m.*)		Chemical profiling of BV (2,2-diphenyl-1-picrylhydrazyl) DPPH assay	
(Nguyen, Yoo, Hwang et al., 2022) [308]	BV (*Apis m.*)		Animal: mice injection Cell line HT22	Cytotoxicity Gene expression pathway Neuroprotection
(Abdelrahaman et al., 2025) [22]	BV (*Apis m.*)	Gentamicin induced kidney injury	Animal: rats Injection	Anti-inflammatory Gene expression-pathway Lipid peroxidation
(Aly et al., 2023) [23]	BV (*Apis m.*)	Epilepsy	Animal: rats Injection Acupuncture	Anti-inflammatory Neurological
(Abu-Zeid et al., 2021) [89]	BV (*Apis m.*)		Animal: rats Injection	Anti-inflammatory Neuroprotective
(Badawi et al., 2020) [26]	BV (*Apis m.*)	Parkinson’s	Animal: mice Injection synergy with L-dopa	Anti-inflammatory Neurological
(Basuini, 2024) [102]	BV (*Apis m.*)		Animal: *Liza ramada* (fish) Characterization of BV [35]	Immunomodulation
(Sobral et al., 2016) [323]	BV (*Apis m.*)		Hela, NCI-H460, Raw264.7, HePG2, MCF-7 cell lines	Anti-inflammatory Cytotoxicity Characterization of BV
(Shaik et al., 2023) [35]	Melittin (honeybee)	Diabetes	Animal: rats Injection Synergy with cordycepin Nanoparticle	Anti-inflammatory Pro-angiogenetic Wound dressing/healing Conjugate
(H.-S. Lee et al., 2021) [90]	BV		Raw264.7, MCF-10A Cell lines	Anti-inflammatory Allergy Cytotoxicity
(Sahsuvar et al., 2023) [159]	Melittin	Cervical cancer	Nsf, MCF-7, C33a, HeLa, 3T3 Cell lines Bacteria: *E. coli* Synergy Conjugate	Anti-bacterial Cytotoxicity Folic acid Melittin hybrid Hemolytic activity
(Tanuğur Samancı & Kekeçoğlu, 2022) [218]	BV		Bee products: Honey Propolis, Royal jelly	Anti-microbial Anti-aging Cytotoxicity Skin care
(Z. Li et al., 2023) [163]	Melittin		Animal: quail gut	Anti-bacterial
(Hegazi et al., 2023) [38]	BV		Acupuncture Clinical	Anti-inflammatory Hemolytic activity Chronic neck pain
(Yaghoubi et al., 2022) [39]	Melittin (*Apis m.*)	Ulcerative colitis	Animal: mice Delivery of melittin Melittin synthesis via fungi	Anti-inflammatory Oxidative stress
(Frangieh et al., 2019) [174]	BV, PLA2 (*Apis m.*)	Breast cancer	MCF-7, 3T3 cell lines Chemical profiling of BV	Anti-bacterial Hemolytic activity
(H. G. Park et al., 2018) [428]	BV elements (*Apis cerana*)			Apoptosis Anti-microbial Cytotoxicity ROS
(Alhage et al., 2018) [429]	PLA2 (*Apis m.*)		DPPH assay	Adverse effects of PLA2
(Elswaby et al., 2022) [227]	BV (honeybee)			Anti-microbial Propolis
(Sani et al., 2022) [430]	Melittin (*Apis m.*)			Membrane interactions Propolis
(H. Jung et al., 2022) [312]	Melittin derived	Cancer	BEAS-2B, RBL-2h3, Raw264.7, HeLa cell lines	Anti-inflammatory Allergy Cytotoxicity
(Nguyen, Yoo, An et al., 2022) [51]	BV		Animal: mice Injection Microneedle delivery of BV	Anti-inflammatory Neuroprotection
(J.-Y. Kim, Lee et al., 2020) [52]	BV (*Apis m.*)	Acute kidney injury	Animal: mice Injection	Anti-apoptotic Anti-inflammatory Oxidative stress
(Orrù et al., 2025) [431]	BV (*Apis m.*)		DPPH assay	
(Mirzavi et al., 2024) [56]	BV (honeybee)	Colon cancer	Animal: mice Injection Xenograft C26 cell line	Anti-inflammatory Anti-tumor Gene expression-pathway
(Senturk et al., 2022) [95]	BV (*Apis m.*)		Animal: rats Injection	Anti-inflammatory Oxidative stress Skeletal muscle, Liver
(Qanash et al., 2025) [322]	BV (*Apis m.*)	Cancer	HePG2 Cell line	Anti-inflammatory, Cytotoxicity, Nanoparticle: Zinc oxide and polyvinyl alcohol
(Lomeli-Lepe et al., 2025) [101]	BV		Acupoint injection Animal: mice	Neuroprotection
(J. Yao, Li et al., 2025) [288]	Not just melittin	Cancer	Animal: rats Topical administration NIH, RAW264.7 cell lines	Synergy with Cu^2+^ skin diseases, ROS

**Table 13:** Experimental design of articles.

Main Focus	Number of Studies				
	In Vivo	In Vitro	In Silico	Clinical	In Vivo and In Vitro
**Anti-inflammatory**	62	29	2	2	NA
**Immunomodulatory**	27	24	1	0	NA
**Anti-microbial**	-	-	-	0	177
**Anti-cancer**	50	127	1	0	NA
**Anti-oxidant**	17	9	0	1	NA

NA, not applicable.
